# Exploring Chemical Diversity of *Phorbas* Sponges as a Source of Novel Lead Compounds in Drug Discovery

**DOI:** 10.3390/md19120667

**Published:** 2021-11-26

**Authors:** Alessia Caso, Fernanda Barbosa da Silva, Germana Esposito, Roberta Teta, Gerardo Della Sala, Laura P. A. Nunes Cavalcanti, Alessandra Leda Valverde, Roberto Carlos C. Martins, Valeria Costantino

**Affiliations:** 1 Department of Pharmacy, University of Naples Federico II, Via D. Montesano, 49, 80131 Naples, Italy; alessia.caso@unina.it (A.C.); germana.esposito@unina.it (G.E.); roberta.teta@unina.it (R.T.); 2 Instituto de Química de Produtos Naturais Walter Mors, Universidade Federal do Rio de Janeiro, Av. Carlos Chagas Filho, 373, Rio de Janeiro 21941-599, Brazil; silvanandab@gmail.com (F.B.d.S.); laurapatricionunes@gmail.com (L.P.A.N.C.); roberto.rcc@gmail.com (R.C.C.M.); 3 Department of Marine Biotechnology, Stazione Zoologica Anton Dohrn, Villa Comunale, 80125 Naples, Italy; gerardo.dellasala@szn.it; 4 Departamento de Química Orgânica, Instituto de Química, Universidade Federal Fluminense, Outeiro de São João Batista s/n, Niterói, Rio de Janeiro 24020-141, Brazil; alessandravalverde@id.uff.br

**Keywords:** *Phorbas*, marine sponges, marine natural products (MNPs), alkaloids, terpenoids, macrolides, cytotoxic metabolites, antimicrobial and anti-inflammatory activities

## Abstract

Porifera, commonly referred to as marine sponges, are acknowledged as major producers of marine natural products (MNPs). Sponges of the genus *Phorbas* have attracted much attention over the years. They are widespread in all continents, and several structurally unique compounds have been identified from this species. Terpenes, mainly sesterterpenoids, are the major secondary metabolites isolated from *Phorbas* species, even though several alkaloids and steroids have also been reported. Many of these compounds have presented interesting biological activities. Particularly, *Phorbas* sponges have been demonstrated to be a source of cytotoxic metabolites. In addition, MNPs exhibiting cytostatic, antimicrobial, and anti-inflammatory activities have been isolated and structurally characterized. This review provides an overview of almost 130 secondary metabolites from *Phorbas* sponges and their biological activities, and it covers the literature since the first study published in 1993 until November 2021, including approximately 60 records. The synthetic routes to the most interesting compounds are briefly outlined.

## 1. Introduction

Biodiversity of marine organisms that reflects on their rich chemical diversity is an important source of novel drug-lead skeletons. Sponges, among other organisms, are one of the main sources of novel skeletons as well as of lead compounds [1,2], promising remedies in drug discovery [3,4] and biotechnological applications. Even if the origin of sponge-derived compounds is still under debate, a growing body of evidence suggests that many marine natural products (MNPs) are produced by microorganisms associated with the sponge [5,6]. In fact, multicellular organisms, such as sponges, are now defined as “holobionts”, i.e., an association between the host and its microorganism community. Therefore, studies performed on sponge organic extracts are actually categorized as studies on holobiont organic extracts [7]. One of the most common classes of sponge-symbionts is cyanobacteria, known as the real producer of some classes of secondary metabolites, specially cyanotoxins [8,9,10].

Chemical diversity coming from marine sponges may still drive drug discovery research [11,12,13]. The genus *Phorbas* is a suitable example to illustrate this point. *Phorbas*-derived natural products discovered so far include compounds belonging to four main classes: alkaloids, macrolides, steroids, and terpenoids. Many of them possess unique structures that might play an important role in biotechnological and pharmaceutical applications.

**Figure 1:**
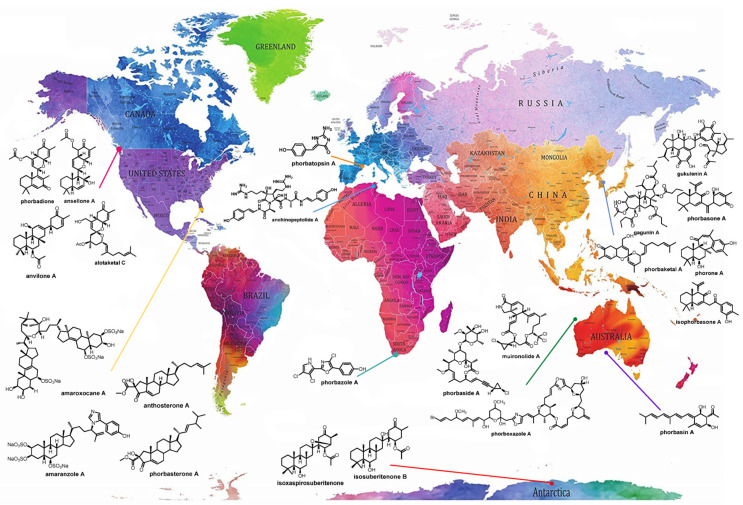
Global distribution of sponges of the genus *Phorbas* and their bioactive metabolites, allowing visualization of the wide distribution of sponges of this genus across all continents, even in the presence of different biotic and abiotic factors. Many metabolites of the same class are found in species collected from different locations. Original world map image source: https://www.amazon.it/Stylish-Living-Mappamondo-Planisfero-Decorazione/dp/B07N1NNPD8 (accessed on 25 February 2021).

The genus *Phorbas* belongs to the class Demospongiae, order Poecilosclerida, family Hymedesmiidae [14], and is the most representative among the 10 accepted genera, which also includes *Hamigera, Acanthancora,* and *Hemimycale* [15,16,17,18]. *Phorbas* stands out not only in the number of isolated MNPs, but also in the large number of bioactive compounds, mainly displaying cytotoxic activity [19]. These sponges are widespread and are present on all continents, including Antarctica (Figure 1) [20]. 

The first study was carried out by Casapullo et al. in 1993, reporting on the structural elucidation of four dimeric peptide alkaloids, known as anchinopeptolides [21,22], from a sample of *Phorbas tenacior* collected along the Tunisian coast. The following year, Rudi et al. (1994) analyzed a sample from South Africa and highlighted, for the first time, the oxazole moiety as an uncommon feature of secondary metabolites from marine sponges [23]. This chemical feature is a distinctive marker of this species, as it is part of phorboxazoles and macrolides, isolated by Searle and Molinski (1995), from a sample collected in Western Australia [24], and of their minor analogue, hemi-phorboxazole A [25]. Secondary metabolites belonging to the alkaloid class have been also isolated from a sample collected along the coast of Marseille, France, and studied by the Bourguet’s group. With this regard, phorbatopsins are alkaloids with a benzylidene 2-aminoimidazolones structure [26]. Finally, the alkaloid-moiety was also present in the structure of a series of imidazolyl steroids, i.e., amaranzoles, isolated from a sample collected in the shallow coral reef water of Key Largo, Florida [27,28].

Nitrogen incorporation seems to be a feature of the *Phorbas* sponge holobiome. Nitrogen is also found in some MNPs belonging to the class of terpenes. Indeed, the taurine unit, not very common in MNPs from sponges, has been found as a part of the terpenes gukulenin E and phorbasins D–G [29,30,31].

This review considers the potential of sponges of the genus *Phorbas* as sources of natural products, and summarizes what is known about their bioactivities. Secondary metabolites belonging to the above-mentioned four classes have been evaluated mainly for cytotoxic activity. However, sponges of the genus *Phorbas* have also been reported as a source of compounds presenting promising antimicrobial, antioxidant, and isocitrate lyase (ICL) inhibition activities [14]. This work provides an overview of the literature records upon *Phorbas* secondary metabolites and their biological activities, starting with the first study published in 1993 until November 2021. 

## 2. Chemistry

### 2.1. Alkaloids

#### 2.1.1. Anchinopeptolides 

**Figure 2:**
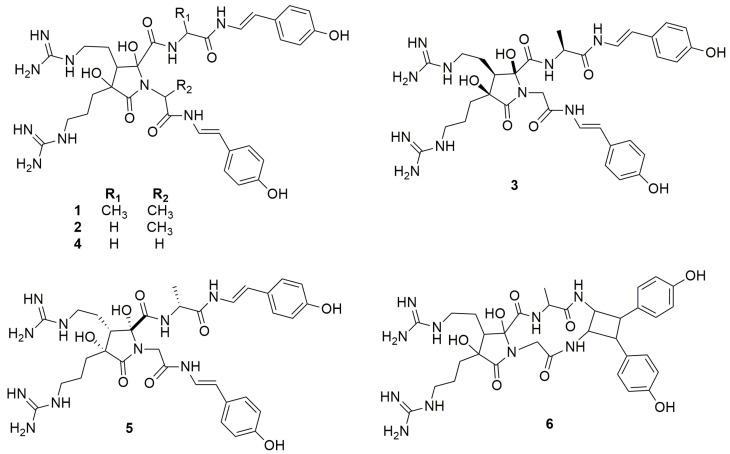
Chemical structures of Anchinopeptolides A–E (**1**–**5**) and Cicloanchinopeptolide C (**6**).

Anchinopeptolide A (**1**) is a dimeric peptide alkaloid isolated from *Phorbas tenacior* (previously *Anchinoe tenacior*) and collected in Tunisia. This is the first report about *Phorbas*, which was published in 1993 [21]. Following this work, Casapullo et al. (1994) described four closely related compounds from the same sponge, the anchinopeptolides B–D (**2**–**4**) and cycloanchinopeptolide C (**6**), formed by an intramolecular cycloaddition of anchinopeptolide C (**3**) [22]. Compound **6** was not detected in the sponge extract but could be synthesized by a photoinduced intramolecular [2+2] cycloaddition. This evidence strongly supports the hypothesis of its artefactual origin [32]. Silva et al. (2019) isolated anchinopeptolide E (**5**), the epimer at C-5′ of compound **3** (Figure 2), from a specimen of *P. tenacior* collected in France. 

**Figure 3:**
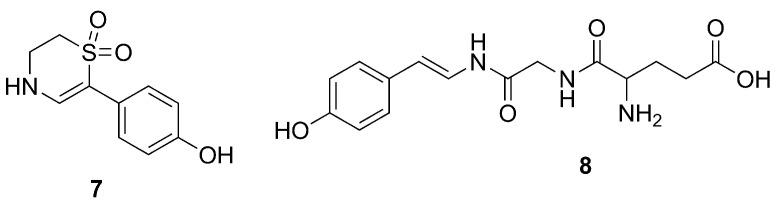
Chemical structures of compounds **7** and **8**.

Further studies on *P. tenacior* allowed the identification of two compounds, i.e., 6-(*p*-hydroxyphenyl)-2H-3,4-dihydro-1,1-dioxo-1,4-thiazine (**7**) and l-glu-gly-4-hydroxystirylamine (**8**) (Figure 3) [33].

#### 2.1.2. Phorbazoles 

**Figure 4:**
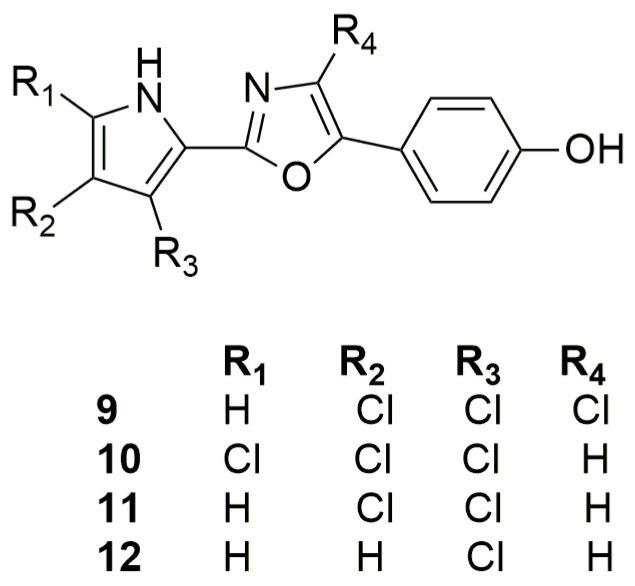
Chemical structures of Phorbazoles A–D (**9**–**12**).

The pioneering work of Rudi et al. (1994) allowed the first isolation of phorbazoles A–D (**9**–**12**) (Figure 4). They represent a new class of marine alkaloids embodying the chlorinated pyrrole moiety. Phorbazoles, isolated in small amounts from a sample collected in South Africa, differ themselves only by the presence and/or position of the chlorine atoms, and their structures were elucidated using mono- and bi-dimensional NMR data coupled with X-ray diffraction studies [34].

In 2020, the total synthesis of the trichlorinated phorbazole B (**10**) has been realized in six steps (overall yield of 23%) using an oxazole as precursor and late stage electrophilic chlorination as key mechanism [35], while a synthesis of phorbazole C was reported in 2001 and relied on pyrrole as a precursor [36]. 

#### 2.1.3. Zarzissine 

**Figure 5:**
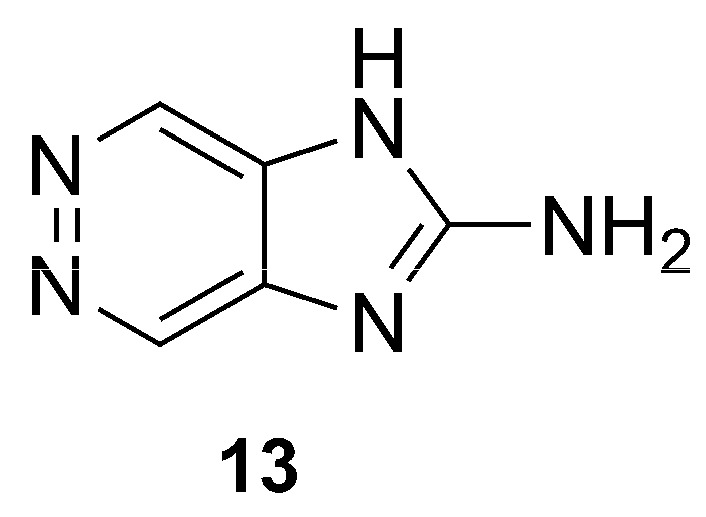
Chemical structure of Zarzissine (**13**).

Among the pioneering works, the study of Bouaicha et al. allowed the isolation of zarzissine (**13**), a pyridazine derivative (Figure 5). This small, highly nitrogenated alkaloid was isolated from a *P. paupertas* (previously *Anchinoe paupertas* and *P. paupertas*) specimen, collected in the Mediterranean Sea, Tunisia, through Sephadex LH-20 column chromatography and preparative HPLC, being unequivocally characterized by GC-MS and NMR analysis [37]. Vacelet and Pérez revised the taxonomy of *P. paupertas*, and the name *Phorbas topsenti* was proposed in 2008 [38]. 

#### 2.1.4. Phorbatopsins

**Figure 6:**
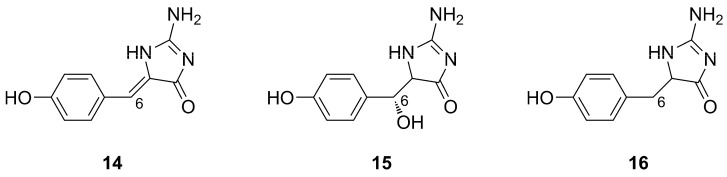
Chemical structures of Phorbatopsins A–C (**14**–**16**).

Phorbatopsins A–C (**14**–**16**) were isolated from *P. topsenti* collected off Marseille at a depth of 5 m, displaying an unprecedented 2-aminoimidazole core (Figure 6). The structure of phorbatopsins was easily elucidated through MS and mono- and bidimensional NMR analysis. The absolute configuration at C-6 of phorbatopsin B (**15**) was determined by using a modified procedure of Mosher’s method [26]. 

Phorbatopsins only differ in the degree of oxidation at C-6. Carbonyl or hydroxyl groups are known for their affinity for free radicals, and the double bond seems to be essential for good antioxidant activity. Biological data perfectly reflect this observation, since compound **14** showed the best activity as a radical scavenger in the ORAC assays, followed by compounds **15** and **16**, that lack both the double bond and the hydroxyl group at position 6 [26].

### 2.2. Macrolides

#### 2.2.1. Phorboxazoles

**Figure 7:**
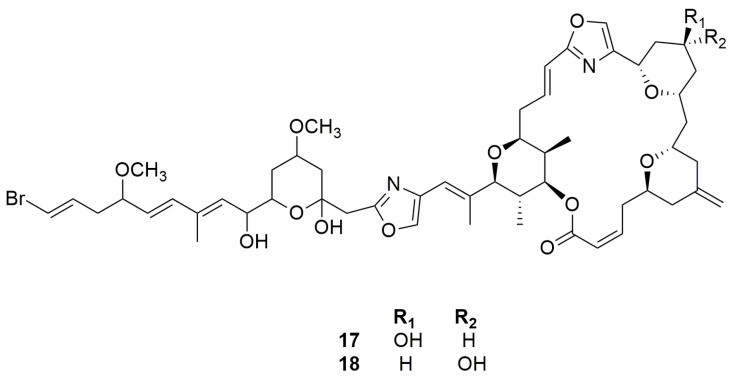
Chemical structures of Phorboxazoles A and B (**17**–**18**).

In 1995, Searle and Molinski [24] reported, for the first time the isolation of two phenyl-pyrrolyloxazoles, phorboxazoles A–B (**17**–**18**) (Figure 7), from a sample collected in western Australia. These compounds belong to the macrolide class containing seven rings, including two oxazoles and four oxane rings, and represent the first members of a novel class of macrolides. Determination of conformation and relative configurations was not an easy task because of the fifteen stereogenic centers and seven rings present in their structure. Nevertheless, this is a didactic example showing how conformation and relative configuration assignment in large macrolide-rings is feasible, providing that some constraints are imposed, such as those due to the presence of oxane and oxazole rings along the macrolide’s perimeter [39]. 

#### 2.2.2. Muironolides

**Figure 8:**
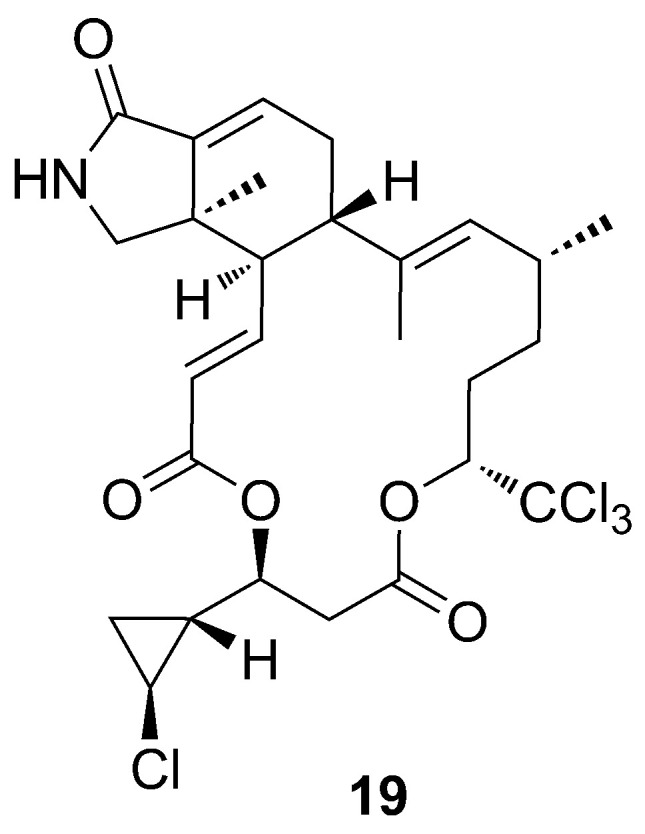
Chemical structure of muironolide A (**19**).

One more example of the incredible *Phorbas* chemodiversity is the isolation of muironolide A (**19**), a novel macrolide, bearing two unprecedented structural features: an hexahydro-1H-isoindolone-triketide ring and a trichlorocarbinol ester (Figure 8) [40].

Muironolide A (**19**) contains a cyclopropane ring, a structural motif already found in phorbasides. Interestingly, the configuration of the cyclopropane ring is opposite compared to phorbaside A (**20**), but the same as that of callipeltoside A, a cytotoxic glycoside macrolide isolated by Zampella et al. (1996) from the new Caledonian sponge *Callipelta* sp. [41].

**Scheme 1:**
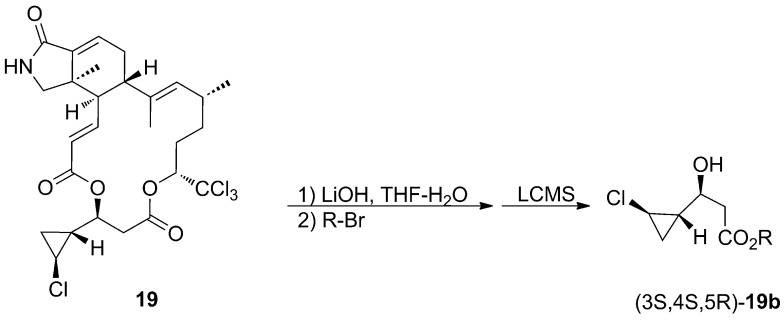
Microscale degradation of Muironolide A (**19**).

The absolute configuration of the macrolide ring has been determined by CD spectroscopy, while the assignment of the absolute stereochemistry of the cyclopropane ring is a nice example of microscale degradation as a useful tool for natural product elucidation, when other powerful methods, such as NMR NOE data and *J*-based assignments, fail [42,43]. Molinski’s group succeeded in the microscale degradation of muironolide A (**19**) with basic hydrolysis to achieve the cyclopropane-unit derivative (Scheme 1).

**Scheme 2:**
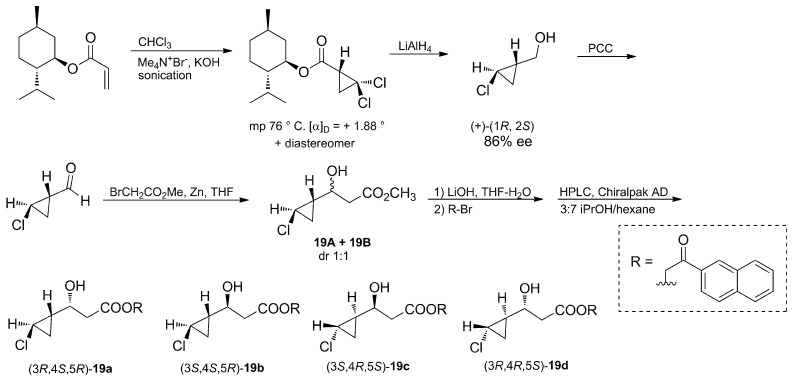
Degradation of (−)-*O*-menthyl acrylate afforded compounds **19a**–**d**, used as standards for the establishment of the absolute stereochemistry of Muironolide A (**19**).

The four possible stereoisomers were synthesized, starting from (−)-*O*-menthyl acrylate (Scheme 2), and used as standards to assign the stereochemistry of the cyclopropane unit of muironolide A (**19**) by comparison of their retention times using chiral LC-MS.

#### 2.2.3. Phorbasides

**Figure 9:**
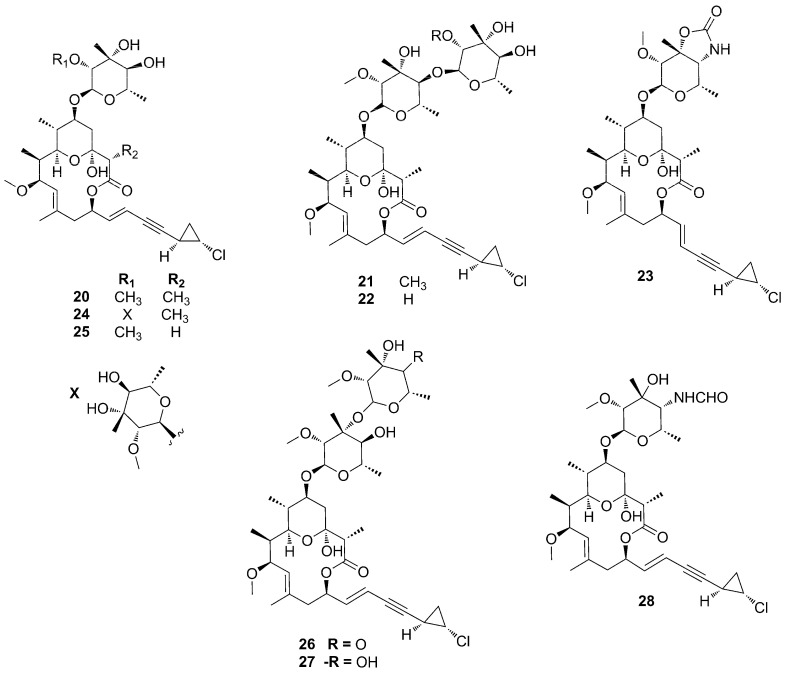
Chemical structures of Phorbasides A–I **(20**–**28**).

Phorbasides (**20**–**28**) are glycosylated macrolides possessing a unique structural motif: an ene-yne-*trans*-2-chlorocyclopropane moiety. They contain the same macrolide ring but different sugar units (Figure 9). Particularly, in phorbaside D (**23**), the sugar unit is fused with an oxazolone unit, while phorbaside B (**21**) and C (**22**) have a 1,4-linked disaccharide made of l-evalose [44,45].

Phorbasides A–E (**20**–**24**), isolated from a *Phorbas* specimen collected off the Western Australian coastline, which also provided phorboxazoles A–B (**17**–**18**), showed different cytotoxicity, which seems to be dependent upon the presence of a free hydroxyl group at position 2′ or 2′ [44]. The repurification of minor fractions from the same sponge extract yielded phorbaside F (**25**) [46], only differing from phorbaside A (**20**) by the absence of a methyl group on the eastern side of the macrolide ring. Phorbaside G (**26**) is the only member of phorbasides family containing a C-4 oxidized pyranoside, while phorbaside I (**28**) is characterized by the presence of a formamide group instead of a hydroxyl group on the pyranoside unit (Figure 9) [47].

### 2.3. Steroids

#### 2.3.1. Phorbasterones 

**Figure 10:**
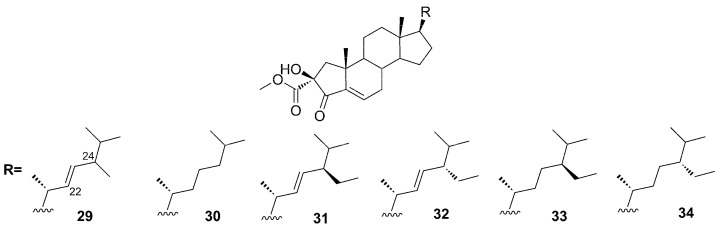
Chemical structures of Phorbasterones A (**29**), B (**30**), Ca (**31**), Cb (**32**), Da (**33**), and Db (**34**).

Mazuno et al. reported, for the first time, the steroids phorbasterones A–D (**29**–**34**) from *Phorbas amaranthus*, collected in Florida [48]. These structures share a characteristic ring A-contracted steroid nucleus and differ from each other by the side chain: phorbasterone A (**29**) possesses a double bond at C-22 and a methyl group at C-24, phorbasterone B (**30**) has a fully saturated isoprenyl side chain, phorbasterone Ca and Cb (**31**–**32**) keep the double bond at C-22 and bear an ethyl group with opposite configuration at C-24, and finally, phorbasterone Da and Db (**33**–**34**) lack the double bond at C-22 but keep the ethyl group at C-24 (Figure 10). 

#### 2.3.2. Anthosterones

**Figure 11:**
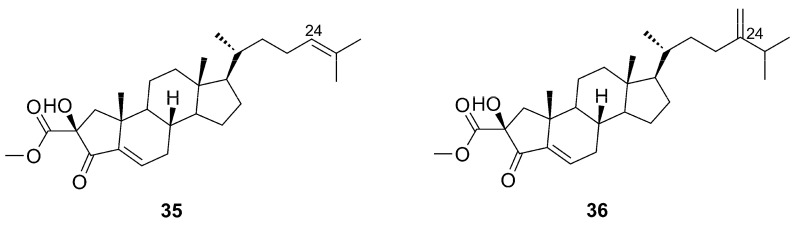
Chemical structures of Anthosterones A–B (**35**–**36**).

Although anthosterones A–B (**35**–**36**) have been isolated from *Anthoarcuata graceae* by Tishler et al. (1988) for the first time [49], they were also isolated later from the organic extract of *Phorbas amaranthus* by Masuno et al. (2004), along with their congeners phorbasterones A–D (**29**–**34**) [48]. Anthosterones A–B (**35**–**36)** (Figure 11) represented the first examples of ring A-contracted steroids displaying a one-carbon appendage at C-2 of the cyclopentane ring. The difference between the compounds resides in the side chain: anthosterone A (**35**) possesses a double bond at C-24 as part of the side chain while anthosterone B (**36**) has an additional olefinic methylene at C-24.

#### 2.3.3. Amaroxocanes 

**Figure 12:**
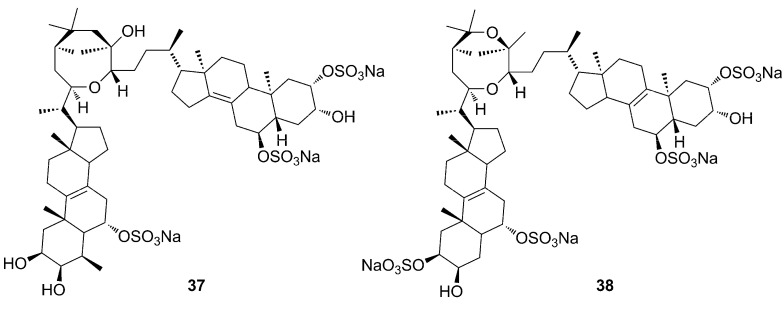
Chemical structures of Amaroxocanes A–B (**37**–**38**).

Morinaka et al. (2009) reported amaroxocanes A and B (**37**–**38**), two dimeric sulfated steroids isolated from *Phorbas amaranthus* collected on shallow coral reefs off Key Largo, Florida [50]. According to Nahar and Sarker (2012), these compounds are characterized by two steroid moieties connected through an oxocane bridge (Figure 12), whose origin comes from different oxidative fusions of the side chains [51].

#### 2.3.4. Amaranzoles 

**Figure 13:**
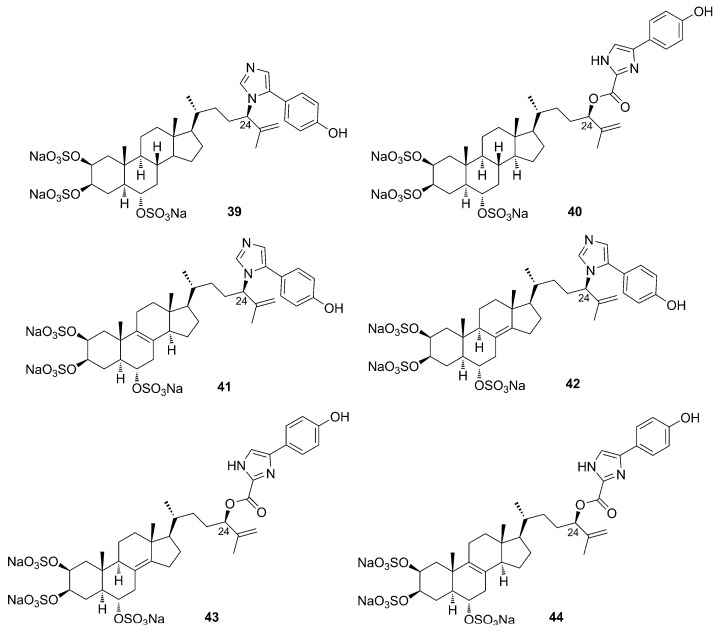
Chemical structures of Amaranzoles A–F (**39**–**44**).

Amaranzole A (**39**) is the first imidazolyl steroidal alkaloid isolated from the tropical sponge *Phorbas amaranthus,* collected in Florida [27]. Extensive studies on the polar extracts of *P. amaranthus* led to the isolation, in 2010, of five additional steroidal alkaloids, amaranzoles B–F (**40**–**44**) [28]. Spectroscopic analysis allowed the structural elucidation of this family of compounds, with the main structural difference consisting of the replacement of the C24-*N*-(4-p-hydroxyphenyl)imidazol-5-yl function with a C24-*O*-(4-p-hydroxyphenyl)imidazole-2- carboxylate motif (Figure 13). 

The authors suggest that the main structural difference in the amaranzole family may be due to an allylic rearrangement that interchange C-24-*N* and C-24-*O* bonds with concomitant loss of CO_2_. The absolute configuration of the side chain at C-24 was assigned through the synthesis of a model ester and the chiroptical comparison of its CD spectrum with that of an amaranzole B (**40**) derivative [28].

### 2.4. Terpenoids

#### 2.4.1. Sesterterpenoids

##### Phorbaketals

**Figure 14:**
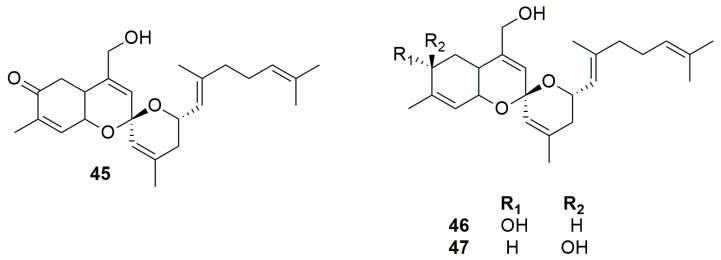
Chemical structures of Phorbaketals A–C (**45**–**47**).

In search of bioactive compounds from sponges of the genus *Phorbas* collected in South Korea, Rho et al. (2009) identified a new type of tricyclic sesterterpenoids, phorbaketals A–C (**45**–**47**) (Figure 14), unprecedented in natural products [52]. They have a unique structure, with a spiroketal ring containing a hydrobenzopyran moiety. Phorbaketal A (**45**) has a carbonyl function at C-5, while phorbaketals B (**46**) and C (**47**) both possess a hydroxyl group in ring A at C-5, but with opposite configuration. The absolute configuration of **45** was determined by chemical conversion. Phorbaketal B (**46**) and C (**47**) are diastereomers and probably originate from a reductive process of phorbaketal A (**45**). Compound **45** has been isolated in large amounts, raising the question of whether its real producer could be a symbiont organism of the sponge. To confirm this hypothesis, the mixed microbial cultures obtained from the homogenized sponge were further cultivated and then re-extracted. As expected, phorbaketal A (**45**) was identified and isolated from the extract of the mixed bacteria culture. This allowed Rho and coworkers to suppose that compound **45** is produced by a symbiotic microorganism [52].

**Figure 15:**
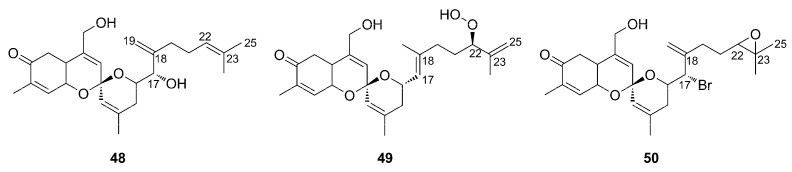
Chemical structure of Phorbaketals L–N (**48**–**50**).

Some years later, three additional members of this family, phorbaketals L–N (**48**–**50**), were elucidated by HRFABMS and NMR analysis from a specimen of *Phorbas* sp. collected in South Korea [53]. Phorbaketals L–N (**48**–**50**) share the tricyclic moiety with the carbonyl group at C-5 with phorbaketal A (**45**), but differ in the side chain at C-16 (Figure 15). While phorbaketal A (**45**) has a double bond at C-17 (Figure 14), phorbaketal L (**48**) differs for the position of this double bond (which is present between C-18/C-19) and for the presence of a hydroxyl group at C-17. Contrariwise, phorbaketal M (**54**) keeps the double bond at C-17 but shows the presence of a hydroperoxide group at C-22 and a double bond at C-23/C-25. Finally, phorbaketal N (**50**) shares the double bond at C-18/C-19 with compound **48** but possesses a bromine instead of a hydroxyl group at C-17; moreover, it possesses an epoxide ring at C-22/C-23. 

Circular dichroism (CD) analysis defined the absolute stereochemistry, suggesting that phorbaketals L–N (**48**–**50**) possess the same absolute configurations as phorbaketal A (**45**).

**Figure 16:**
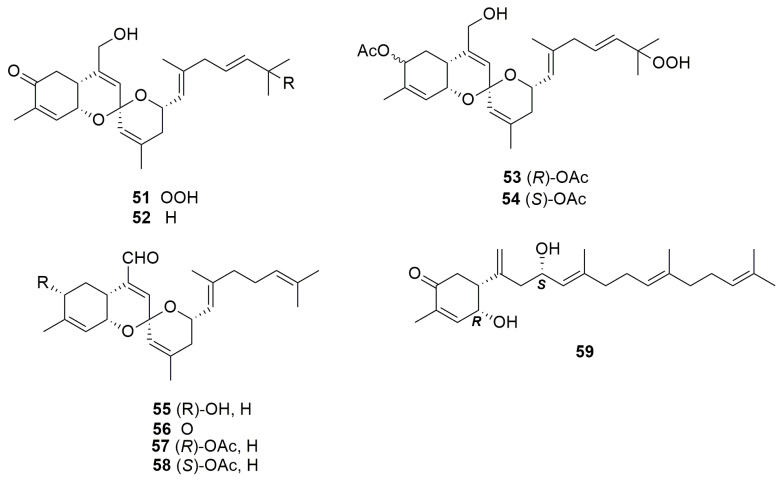
Chemical structures of Phorbaketals D–K (**51**–**58**) and phorbin A (**59**) isolated from sponge *Monanchora* sp.

Wang et al. (2013) isolated eight new sesterterpenoids, namely phorbaketals D–K (**51**–**58**), from the organic extract of the Korean marine sponge *Monanchora* sp., along with their progenitors phorbaketals A–C (**45**–**47**), and phorbin A (**59**), a possible precursor of these sesterterpenoids (Figure 16) [54]. These compounds have not yet been identified in sponges of the genus *Phorbas*.

##### Alotaketals

**Figure 17:**
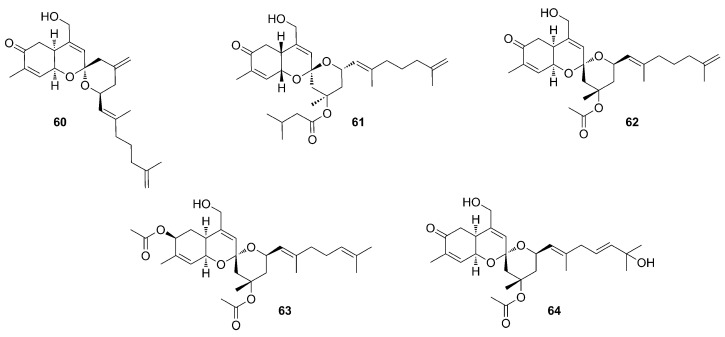
Chemical structures of Alotaketals A–B (**60**–**61**) isolated from *Hamigera* sp., and Alotaketals C–E (**62**–**64**) isolated from *Phorbas* sp.

Aloketals are sesterterpenoids with an unprecedented alotane carbon skeleton. Aloketal A (**60**) and B (**61**) (Figure 17) were isolated from the extracts of the sponge *Hamigera* sp., collected in Papua New Guinea [55]. They are the first members of a family of sesterterpenoids that may have the same “alotane” precursor, like other compounds identified in *Phorbas,* such as phorbaketals (**45**–**47**) and suberitonones (**80**–**85**). Alotane sesterterpenoids have been isolated from sponges collected in three widely separated locations around the Pacific Rim (Papua New Guinea, Korea, and British Columbia). Alotaketal C (**62**) was isolated by Daoust et al. (2013) from a specimen of *Phorbas* collected in British Columbia (Figure 17) [56]. 

Further studies aimed at an exhaustive search for minor sesterterpenoid compounds in the same *Phorbas* extract yielding alotaketal C (**62**) allowed the identification of alotaketals D (**63**) and E (**64**) (Figure 17) [57]. 

The basic skeleton of alotaketals is a tricyclic structure, very similar to phorbaketals (**45**–**58**). Alotaketals possess a carbonyl group at C-4 in ring A, except for alotaketal D (**63**), in which the carbonyl is substituted by an acetoxy function [57]. 

The unique characteristics of the structures of phorbaketals (**45**–**58**) and alotaketals (**60**–**64**) have inspired many synthetic studies that have been recently reviewed [58]. Here, we outline the recent effort of Tong et al. that accomplished an asymmetric total synthesis of (−)-phorbaketal A (**45**) and (−)-alotaketals A–B (**60**–**61**) by a novel approach based on the cyclization of vinyl epoxy δ- keto-alcohols [59]. It is worth noting that the tricyclic spiroketal tricycle intermediate was obtained on an impressive 4.5 g scale. To complete the synthesis, the installation of the terminal alkene allowed the late-stage introduction of various side chains via cross-metathesis reaction (the corresponding aldehyde olefination strategy was unsuccessful), allowing access to five structurally different tricyclic spiroketal alotane-type sesterterpenoids. Moreover, in 2020, Lee and coworkers [60] reported on a novel gold-mediated spiroketalization of an epoxy alkyne followed by the rearrangement into the corresponding allylic alcohol being applied to the total synthesis of (−)aloketal A, carried out through 11 steps from 4-hydroxycarvone. 

**Scheme 3:**
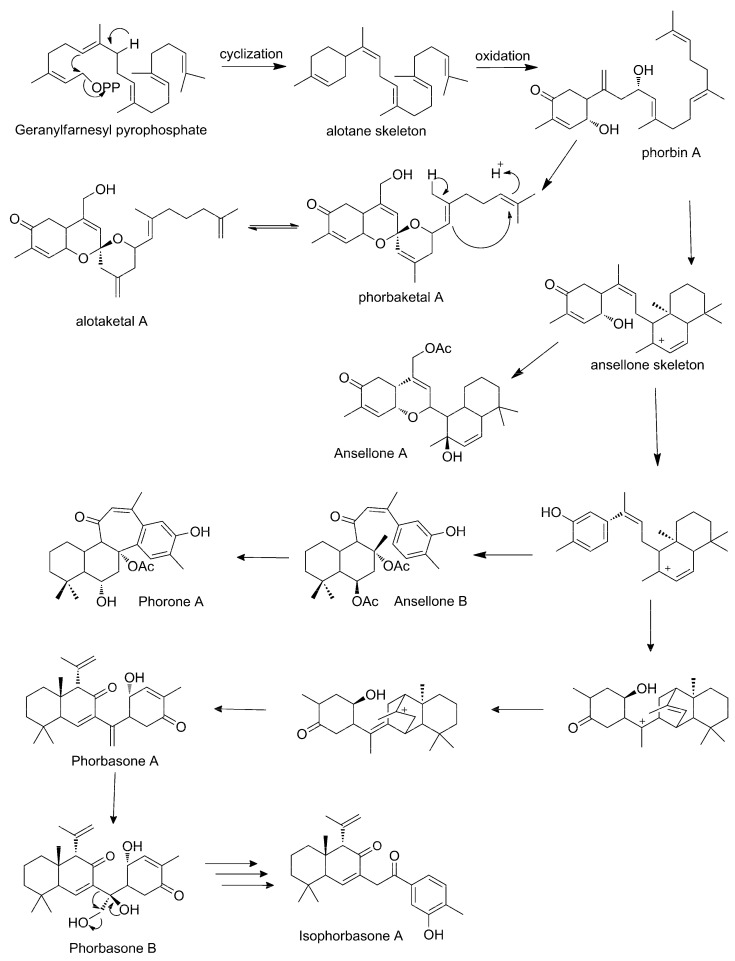
Proposed biosynthesis of sesterterpenoids of *Phorbas* (adapted from Wang et al., 2012).

Some authors also propose a possible biosynthetic route for the formation of these natural products (Scheme 3, paragraph 5.1.8) [61].

##### Ansellones

**Figure 18:**
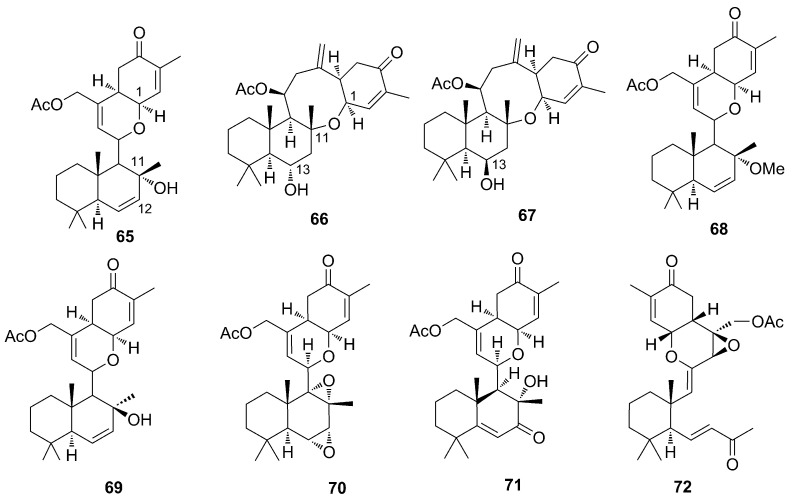
Chemical structures of Ansellones A–G (**65**–**71**) and Secoepoxyansellone A (**72**).

The first member of the ansellone sesterterpenoid family is ansellone A (**65**) (Figure 18) from the nudibranch *Cadlina luteomarginata* and the sponge *Phorbas* sp., collected in British Columbia, Canada [62]. Compound **65** was identified as the main component in the organic extract from both organisms. It has an unprecedented ansellane tricycle skeleton. This new group of sesterterpenoids is biogenetically related to alotaketals and phorbaketals [62,63]. Through HRESIMS and NMR analyses, it was possible to identify the tetracyclic structure of ansellone A (**65**) and the positions of its double bonds and ring substituents. Ansellone A (**65**) is sequestered by *C. luteomarginata* from a *Phorbas* sp. diet [62].

As previously mentioned, in the 2013 study by Daoust et al., where alotaketals were identified, ansellone B (**66**) and secaepoxyansellane A (**71**) were also identified [56]. Following this work, Wang and coworkers (2016) identified ansellones D–G (**67**–**70)** (Figure 18) [57].

The ansellones (**65**–**70**) are tetracyclic sesterterpenoids. The secoepoxyansellone A (**72**) has three six-membered rings, one of them linked to an epoxide. Ansellones B (**66**) and C (**67**), epimers at C-13, feature an unprecedented heterocyclic skeleton that contains an oxocane ring [56]. Ansellone A (**65**) and E (**69**) are epimers whose difference lies in the configuration at C-11; ansellone D (**68**) has a methoxy instead of a hydroxy group in the same orientation as ansellone A (**65**). Ansellone F (**70**) displays two epoxide functions and ansellone G (**71**) has an α,β-unsaturated carbonyl function [57]. 

Due to its activity as an HIV latency-reversing agent, ansellone A (**65**) has attracted interest and, very recently [64], the total synthesis of ansellone A (**65**) and B (**66**) and analogues has been reported [63]. The key reaction proposed by Tong and coworkers is a Prins cyclization reaction, enabled using the TfO group for stabilization of the acid-labile tertiary allylic alcohol. The asymmetric synthesis of three ansellane-type compounds, i.e., (−)-ansellone A (**65**) and B (**66**) and (+)-phorbadione (**75**), was accomplished in 16–23 steps from the commercially available (+)-sclareolide. SAR studies showed that the alcohol analogue exhibits a better activity than **65**. 

##### Anvilones

**Figure 19:**
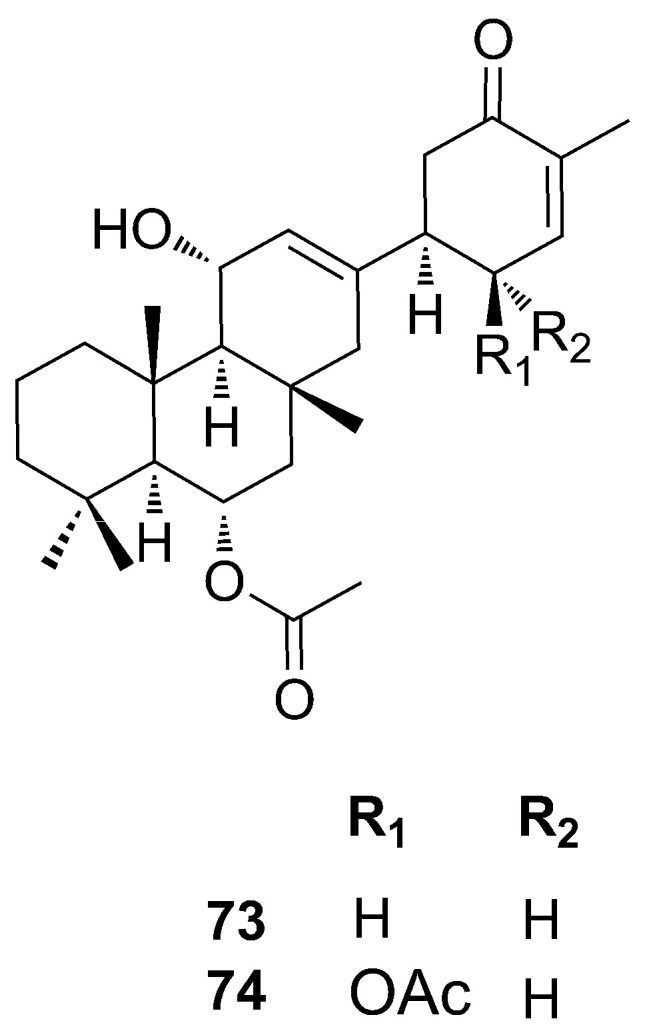
Chemical structures of Anvilones A–B (**73**–**74**).

Anvilones represent another group of sesterterpenoids identified by Wang and coworkers (2016) from *Phorbas* sp. collected in British Columbia, Canada. Anvilones A–B (**73**–**74**) have the unprecedented “anvilane” sesterterpenoide carbon skeleton [57]. They were identified by ESI-MS and NMR and are tetracyclic sesterterpenoids. Anvilone B (**74**) differs from A (**73**) for the addition of an acetoxy functionality (Figure 19).

##### Phorbadione

**Figure 20:**
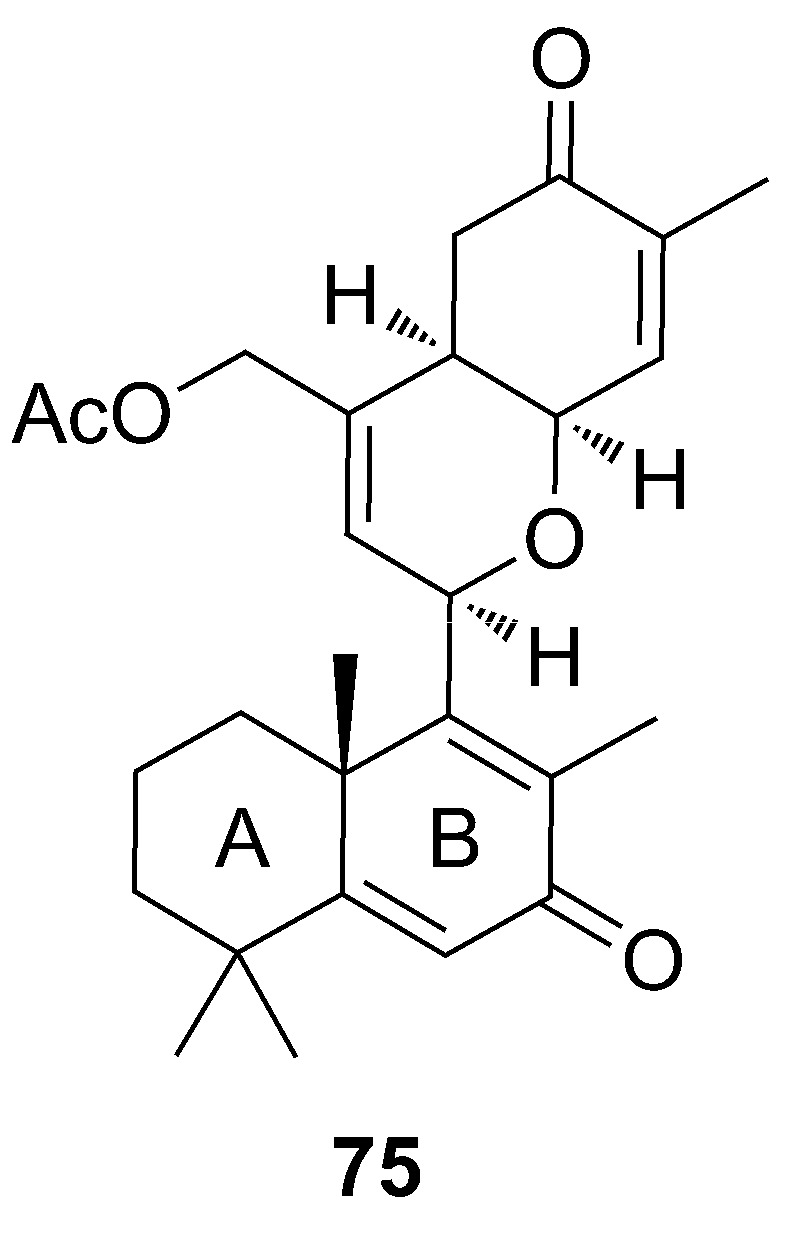
Chemical structure of Phorbadione (**75)**.

The phorbadione (**75**) was isolated by Daoust and coworkers in 2013, together with ansellone B (**66**), secoepoxyansellone A (**72**), and alotaketal C (**62**) from samples of *Phorbas* sp. collected in British Columbia. ESIMS and NMR data of **75** showed great similarity with ansellone A (**65**), due to the presence of the cyclohexenone and dihydropyran rings. Phorbadione (**75**) is different from ansellone A (**65**) due to an extra unsaturation and an α,β-unsaturated carbonyl function in the ring B [56] (Figure 20).

##### Phorbasones

**Figure 21:**
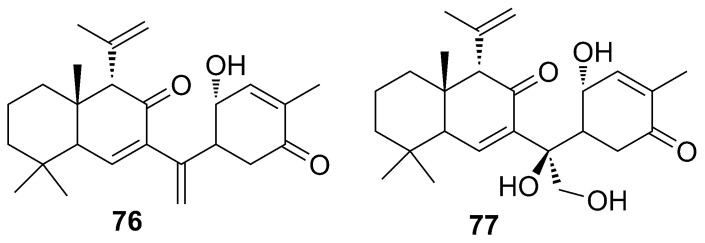
Chemical structure of Phorbasones A and B (**76**–**77**).

Phorbasones A and B (**76**–**77**) (Figure 21) are sesterterpenoids identified together with phorbaketals [65] from a specimen of *Phorbas* sp. collected in Korea. It is likely that ansellones (**65**–**71**), phorbaketals (**45**–**58**), and phorbasones (**76**–**77**) are biogenetically linked (Scheme 3, paragraph 5.1.8). Complete structures of phorbasones A–B (**76**–**77**) were elucidated by spectroscopic techniques and chemical reactions. They have a bicyclic structure connected by a small side chain to a cyclohexanone. The difference between them resides in the bridge-chain, as phorbasone B (**77**) has a hydroxymethylene and an additional hydroxyl group, and phorbasone A (**76**) possesses a methylene function at the same position. The absolute stereochemistry of compounds was determined by the Mosher’s ester method and NMR data. 

**Figure 22:**
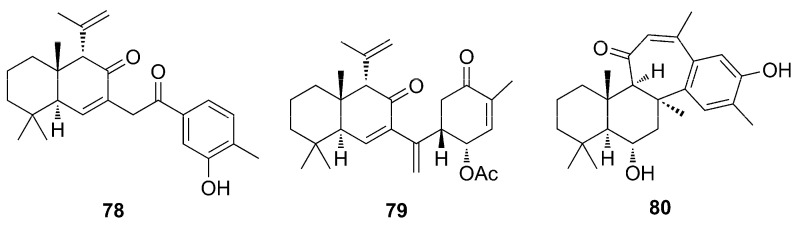
Chemical structures of Isophorbasone A (**78**), Phorbasone A Acetate (**79**), and Phorone A (**80)**.

One year later, a further study carried out with samples from *Phorbas* collected in Korea, and from which ansellone B (**66**) was identified, allowed the identification of a new structure called isophorbasone A (**78**) (Figure 22) [66]. Compound **78** presents the bridge-chain containing one more carbon unit, and the cyclohexanone ring is replaced by phenol when compared to phorbasone A (**76**). In addition, data from this work allowed the identification of phorone A (**80**) (Figure 22), in which the tetracyclic skeleton consisting of a bicyclic system (i.e., decalin, similar to those of phorbasones) is fused to a cycloheptanone ring, fused in turn to a fourth phenyl ring [66]. 

##### Suberitenones

**Figure 23:**
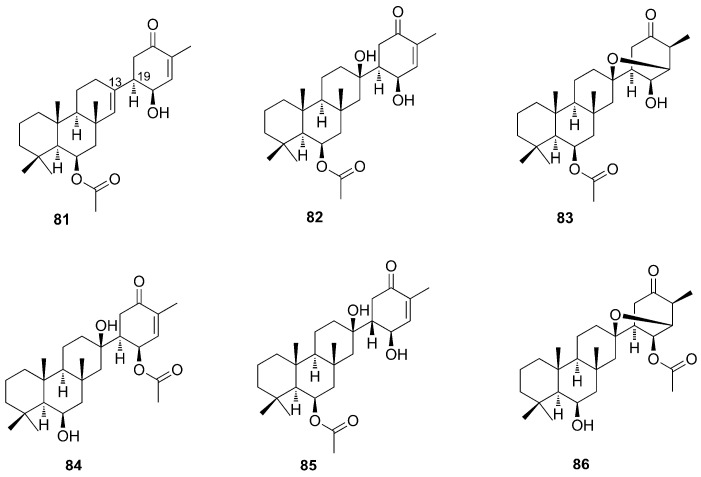
Chemical structures of Suberitenones and natural products derivatives (**81**–**86**).

Sesterterpenoids of the suberitone type were isolated and identified from the sponge *Phorbas areolatus*, collected in Antarctica. The main feature of these substances is the rare suberitane skeleton. In addition to the already known suberitenones A and B (**81**–**82**) and oxaspirosuberitenone (**83**), the new isosuberitenone B (**84**), 19-episuberitenone B (**85**), and isooxaspirosuberitenone (**86**) were isolated (Figure 23) [67]. Until then, these substances were considered markers of the *Suberites* genus, the only one in which they had been identified. The discovery from *Phorbas* excludes suberitanes as chemotaxonomic markers.

This sesterterpenoids were identified based on HRMS and NMR data analysis by comparisons with those of the same group already reported. Suberitenone A (**81**) has a double bond at C-13 absent in B (**82**), which has a hydroxyl at this position. The HRMS data analysis showed that isosuberitenone B (**84**) and suberitenone B (**82**) are isomers. The analysis of ^1^H-NMR and HMBC spectra showed that the difference between the two compounds resides in the position of the acetoxy substituent, which in isosuberitenone B (**84**) is on the cyclohexanone ring, while in suberitenone B (**82**) it is located on ring B. The epimer at C-19 of **82** is 19-episuberitenone B (**85**). As observed for suberitenone B (**82**) and isosuberitenone B (**84**), isooxaspirosuberitenone (**86**) has NMR signals very similar to oxaspirosuberitenone (**83**), with the difference being the position of the acetoxy substituent group (Figure 23). Interestingly, these structures come from a 1,4 nucleophylic addition and cyclization process which involves the alcohol group at C-13 and the enone of the D ring, thus resulting in a rare structure in natural products [67].

##### Putative Biosynthetic Pathway of Sesterterpenoids from *Phorbas*

Some authors have already proposed biosynthetic routes for the formation of sesterterpenoids found in *Phorbas*. As being found in the same species, several of these natural products can share stages on the same route. The main hypothesis is that they all start from the geranyl-farnesyl pyrophosphate, which through cationic cyclizations, oxidation, and rearrangements, would lead to different skeletons [66].

Phorbaketals (**45**–**58**) and alotaketals (**60**–**64**) are compounds with a spiral ring originated from the geranyl–farnesyl pyrophosphate unit. A pyrophosphate-generated allylic cation is attacked by proximal olefin to form the alotane skeleton and, after successive oxidations, it produces phorbin A (**59**), phorbaketal (**45**–**58**), and alotaketal (**60**–**64**) [61]. 

Phorbin A (**59**) is considered a precursor of a series of *Phorbas* steroids, such as phorbasones (**76**–**77**). The farnesyl part of phorbin A (**59**) undergoes cationic cyclization, forming the skeleton of ansellone, a shared intermediate between phorbasones (**76**–**77**), ansellone (**65**–**71**), and phorone A (**80**), which has a common fused bicyclic ring. The ansellone skeleton reorganizes itself through cationic cyclization, forming a bicyclic structure which undergoes successive proton migrations and ring cleavage. This sequence of reactions leads to the tricyclic structure of phorbasone A (**76**), which is hydroxylated to phorbasone B (**77**). For the formation of isophorbasone A (**78**), phorbasone B (**77**) passes through several stages, such as oxidation, dehydration, rearrangement, and tautomerization of the hexenone keto-enol (Scheme 3) [65,66].

**Scheme 4:**
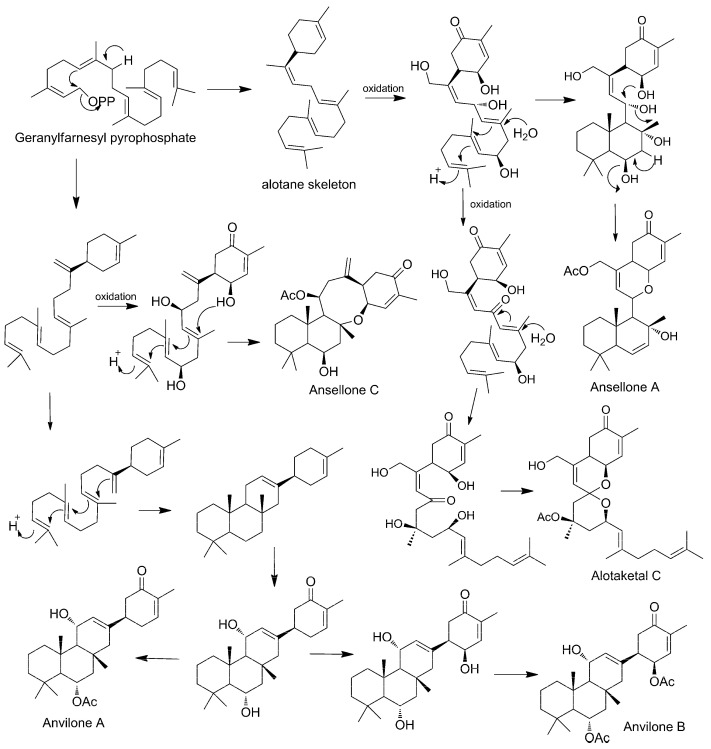
Proposed biosynthesis of sesterterpenoids identified in *Phorbas* (from Wang et al., 2016).

The ansellone skeleton is the precursor of the ansellone (**65**–**71**) class and phorone A (**80**). Wang et al. link the biosynthetic route of the ansellones with that of the anvillones (**73**–**74**) and alotaketals (**60**–**64**), found in *Phorbas* sp. collected in British Columbia (Scheme 4) [57].

#### 2.4.2. Diterpenoids

##### Phorbasins

**Figure 24:**
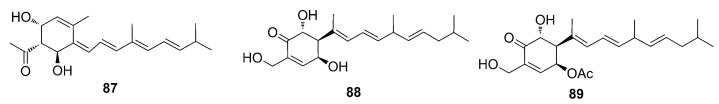
Chemical structures of Phorbasins A–C (**87**–**89**).

In 2000, Vuong and Capon identified a diterpene with a novel skeleton, named phorbasin A (**87**), from a *Phorbas* sp. sample collected in Australia [31]. NMR and ESI-MS analyses allowed the identification of this novel skeleton as a conjugated polyene attached to a monocyclic unit. The molecule was unstable and underwent decomposition during the analysis. Therefore, the absolute stereochemistry of the three chiral centers of phorbasin A (**87**) remains undetermined. The enantiomer portrayed in Figure 24 displays the arbitrary absolute stereochemistry suggested by authors. 

One year later, two additional phorbasins were identified, namely, phorbasin B and C (**88**–**89**) [68]. The relative stereochemistry of phorbasin B (**88**) was attributed based on coupling constant values and molecular modeling data that provided theoretical measures of the dihedral angles. The stereo structure was established by comparison with other similar skeletons having the same cyclohexenone substructure. Phorbasin C (**89**) was spectroscopically very similar to phorbasin B (**88**). The molecular formula for phorbasin C (**89**) differed from phorbasin B (**88**) by 42 mass units (acetyl unit), and the ^1^H NMR spectrum of phorbasin C (**89**) revealed signals related to the presence of a characteristic methyl acetate group. Due to the lack of material and the instability of phorbasin A (**87**) and C (**89**), their absolute stereochemistry could not be determined. One year later, the total synthesis of phorbasin C (**89**) was carried out by Macklin and coworkers, finally allowing the elucidation of the relative and absolute configuration of this phorbasin [69]. The skeleton of phorbasins B–C (**88**–**89**) is very similar to phorbasin A (**87**) (Figure 24). They differ by the side chain and by substituents on the cyclohexenone ring. These diterpenoids are likely to share a common biosynthetic pathway, even if not occurring as co-metabolites [68].

**Figure 25:**
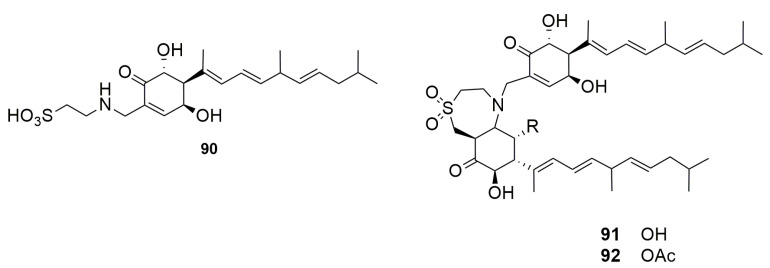
Chemical structures of Phorbasins D–F (**90**–**92**).

Following these two studies, Zhang and Capon (2008) reisolated the phorbasins B–C (**88**–**89**) and confirmed their stereochemistry. Additionally, they identified new analogs named phorbasins D–F (**90**–**92**) (Figure 25), which incorporate a somewhat unprecedented terpenyl-taurine residue (2-aminoethanesulfonic acid) [70]. ESI-MS and NMR analyses allowed the identification of phorbasin D (**90**). The comparison of NMR data of phorbasins B–C (**88**–**89**) and D (**90**) showed the presence of different functional groups on the cyclohexenone ring. While in phorbasins B–C (**88**–**89**) the substituent is a hydroxymethylene, a taurinyl group is present in phorbasin D (**90**). 

Phorbasins E–F (**91**–**92**) are actually dimers, probably derived from phorbasin B or C (**88**–**89**), fused by a seven-membered heterocyclic ring which incorporates a taurinyl residue, an unprecedented feature in natural product skeletons [70]. Analysis of the structures that form the dimers suggested that they may have the same biosynthetic origin and, therefore, a common absolute stereochemistry. The difference between these dimers is the presence of an acetate substituent linked to the phorbasin F (**92**), which is absent in E (**91**) [31,68,70].

**Figure 26:**
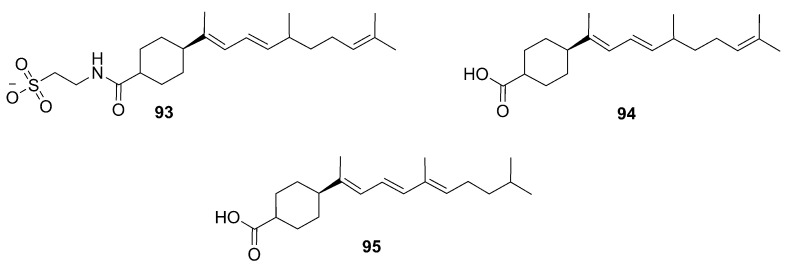
Chemical structures of Phorbasins G–I (**93**–**95**) identified by Lee et al. (2008).

In 2008, Lee and coworkers identified three novel diterpenes from *Phorbas gukhulensis*, collected in South Korea, that were named phorbasins G–I (**93**–**95**) [71] (Figure 26). These molecules, identified by FAB-MS and NMR experiments, feature a cyclohexane ring, in contrast to their previously discussed congeners, which exhibits a cyclohexene moiety. Phorbasin G (**93**) has a taurine residue as a substituent on the ring, very similar to that found in phorbasin D (**90**). Phorbasin H (**94**) displays a carboxylic function, as well as phorbasin I (**95**), which differs from the former by the position of a double bond in the side chain [56]. Recently, the synthesis of phorbasin H (**94**) was carried out and the absolute configuration of the natural product was determined as *S* [72]. A dereplication work through LCMS showed the presence of these phorbasins G–I in the extract of *P. amaranthus* [73].

**Figure 27:**
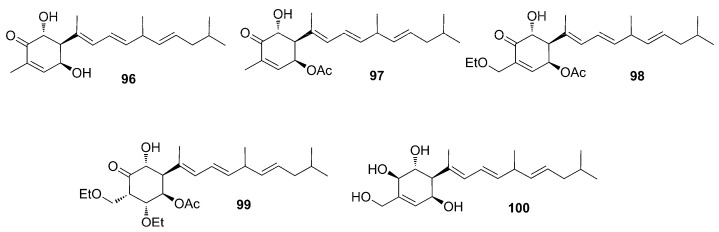
Chemical structures of Phorbasins G–K (**96**–**100**) identified by Zhang and Capon, 2008.

A few months later, Zhang et al. identified five additional phorbasins, i.e., phorbasins G–K (**96**–**100**) (Figure 27) [74]. Analysis of ESI-MS and NMR spectra revealed that phorbasin G (**96**) is a desoxy analogue of phorbasin B (**88**), while phorbasin H (**97**) is an acetate derivative of phorbasin G (**96**) sharing the same absolute stereochemistry. Phorbasin I (**98**) has an ethoxy group at C17, and phorbasin J (**99**) bears two ethoxy groups, respectively at C2 and C17, and so both phorbasins can be regarded as ethoxy derivatives of phorbasin H (**97**). Phorbasin K (**100**) was reported as the dihydro analogue of phorbasin B (**88**). Phorbasins I and J (**98**–**99**) are likely solvolysis artifacts generated by storage in ethanol.

##### Gagunins

**Figure 28:**
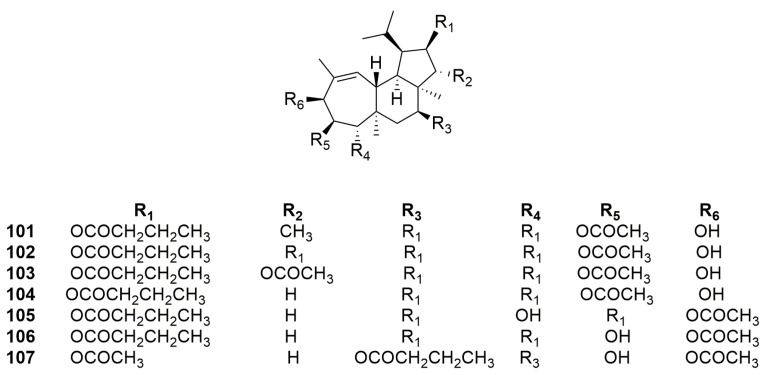
Chemical structures of gagunins A–G (**101**–**107**).

Seven diterpenoids, named gagunins A–G (**101**–**107**) (Figure 28), have been identified [75] for the first time from a species collected in South Korea. They have the core structure of homoverrucosane, with three fused carbon rings, i.e., cyclopentane, cyclohexane, and cycloheptene. The main difference with the core structure of homoverrucosane is the stereochemistry of the junction between the five and six carbon rings. Another feature is that they are highly oxygenated structures with a variety of functions.

The gagunins’ structures were defined through FAB-MS, IR, and NMR data analyses. Structural elucidation of gagunin A (**101**) was achieved by its conversion into a perohydroxyl derivative to remove ester chains, making the structural elucidation easier. Gagunins A–C (**101**–**103**) differ from each other by the two substituents on the cyclopentane ring. Gagunins D–F (**104**–**106**) differ by the three substituents on the cycloheptene ring. Gagunin G (**107**) shares the substitution pattern of the cycloheptene ring with gagunin F (**106**) and differs from the latter by a substituent on the cyclopentane ring [75].

**Figure 29:**
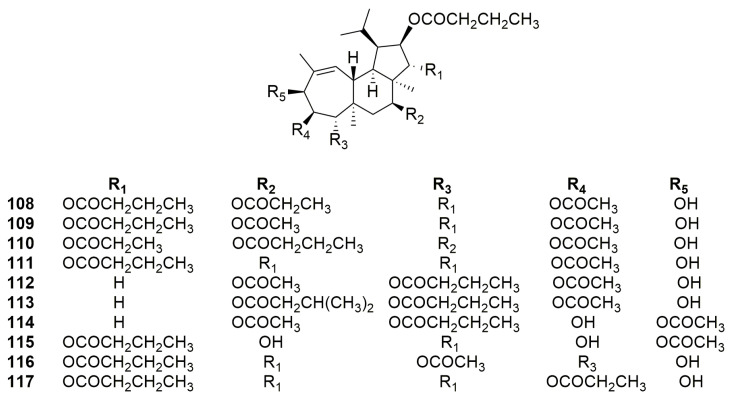
Chemical structures of Gagunins H–Q (**108**–**117**).

In 2008, Jang and coworkers, from the same *Phorbas* sp. sample collected in Korea, identified phorbasin H (**94**), as previously mentioned, and six known gagunins, i.e., gagunins A–D (**101**–**104**) and F–G (**106**–**107**), and ten new gagunins, named H–Q (**108**–**117**) (Figure 29) [76].

Gagunins H–M (**108**–**113**) are very similar to gagunins A–D (**101**–**104**), showing changes in the substitution pattern of the cyclohexane ring differently from compounds **101**–**104**. Gagunins N–O (**114**–**115**) have a close similarity with gagunins F–G (**106**–**107**), but with a different substitution pattern on the cyclohexane ring. Gagunins P–Q (**116**–**117**) have an identical substitution pattern in the cyclopentane and cyclohexane rings, and changes are in the substituent groups on the cycloheptene ring (Figure 29) [76].

#### 2.4.3. Tetraterpenoids

**Figure 30:**
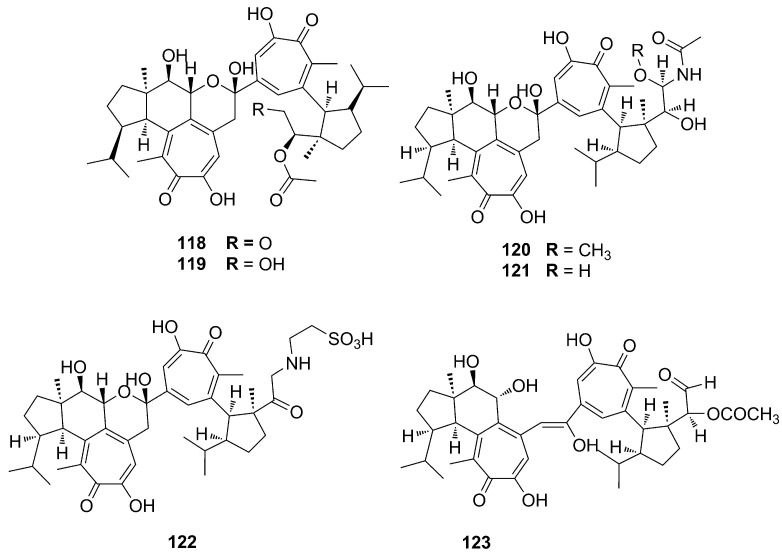
Chemical structures of Gukulenins A–F (**118**–**123**).

To date, the only tetraterpenoids identified from the genus *Phorbas* are gukulenins, which have an unprecedented *bis*-tropolone skeleton. Gukulenins were identified from sample of *P. gukhulensis*, collected in South Korea. Gukulenins A–F (**118**–**123**) look like pseudodimers of the gagunines, also found in this species, but the different positions of substituting groups may indicate that they have a different precursor. They have very diverse oxygenated side chains, but they are smaller than the oxygenated groups found in gagunins. Moreover, they also have aminoacidic chain linked to one of the two tropolone unit [29,30] (Figure 30). 

It is worth mentioning that the synthesis of this dimeric tetraterpenoid structure has been recently explored by D. Tymann and coworkers, who proposed a two-carbon ring expansion photochemical mechanism to get an *alpha*-tropolonic ether ring, starting from (−)-piperitone. The same group states that total synthesis of gukulenin A is under investigation [77].

### 2.5. Miscellaneous 

#### 2.5.1. Other Compounds Identified from *P. topsenti*

**Figure 31:**
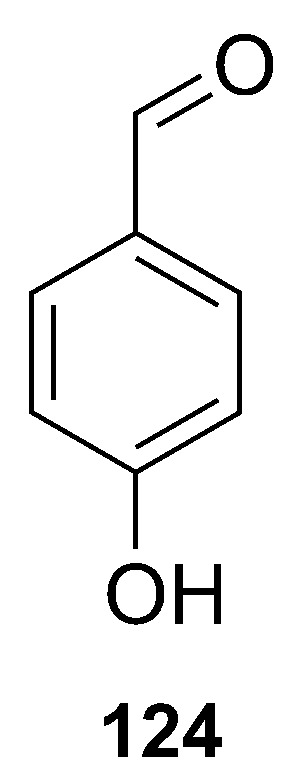
Chemical structure of the *p*-hydroxybenzaldehyde (**124**).

The *p*-hydroxybenzaldehyde (**124**) was identified in the same study where the alkaloid zarzissine (**13**) was isolated (Figure 31). This compound was identified in the sponge *P. topsenti* (previously *P. paupertas*) collected from the Mediterranean Sea off the coast of Tunisia [37].

**Figure 32:**
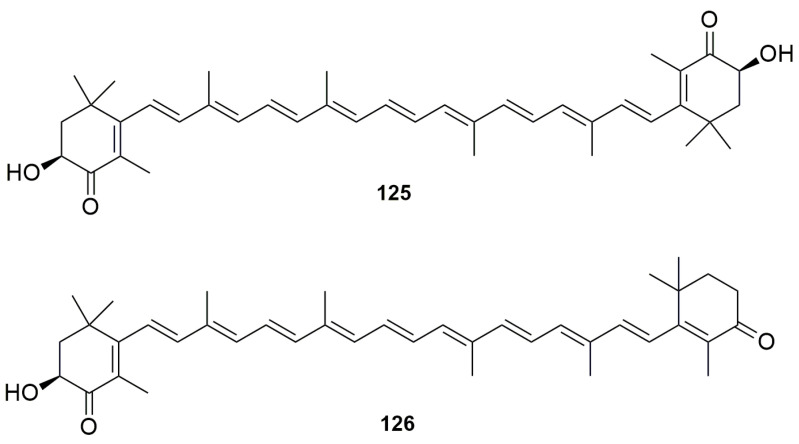
Chemical structures of the Astaxanthin (**125**) and Adonirubin (**126**).

The carotenoids astaxanthin (**125**) and adonirubin (**126**) (Figure 32) were isolated from a species of *Phorbas topsenti* collected off Marseille (France), in addition to the alkaloids phorbatopsins (**14**–**16**) [26]. 

**Figure 33:**
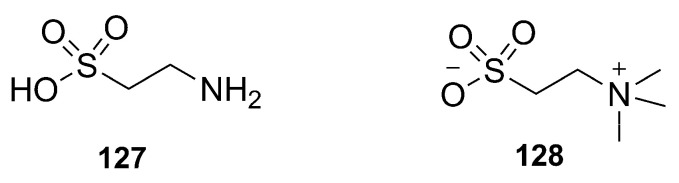
Chemical structures of Taurine (**127**) and Taurobetain (**128**).

In the framework of the study mentioned above, two sulfonic acids, taurine (**127**) and taurobetain (**128**), were isolated (Figure 33) [26].

#### 2.5.2. Other Compounds Identified from *P. amaranthus*

Recently, a dereplication study, based upon comparison of HRMS fragmentation pathways, allowed the annotation of 18 known *Phorbas* metabolites from the extract of *P. amaranthus*, collected in Brazil. Among others, phorbasins (**87**–**88**, **97**–**100**), phorbaketals (**45**, **46** or **47**,**49**), isophorbasone (**78**), ansellones (**72**), anvillones (**80**), and phorbasterones (**29**–**36**) [73] were isolated. 

**Figure 34:**
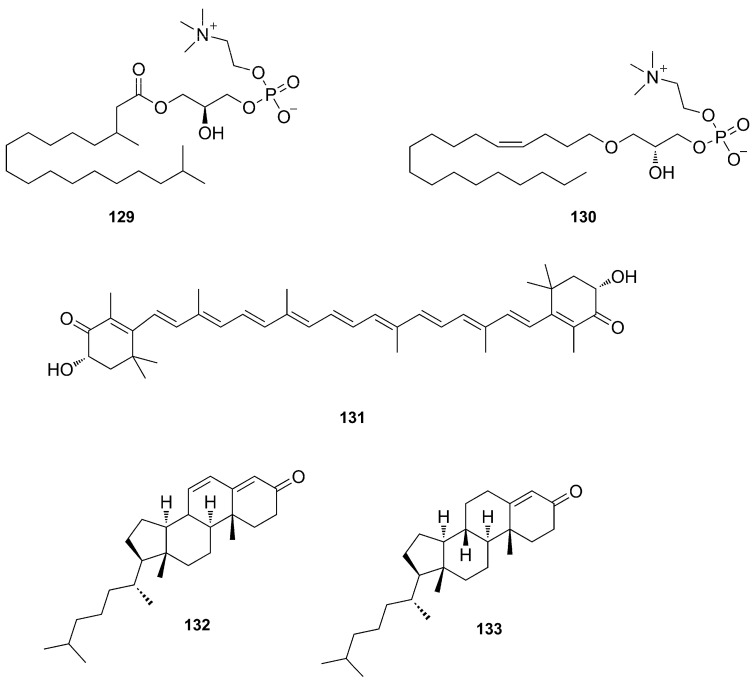
Chemical structures of compounds detected by GNPS in the organic extract *P. amaranthus* (**129**–**133**).

Bioinformatic analyses of MS/MS data (Global Natural Product Social Molecular Networking (GNPS) [78] of the same extract allowed the identification of 17 additional metabolites, most of them being lysophospholipids (LPL) (**129**–**130**), carotenoids (**131**), and sterols (**132**–**133**) (Figure 34). The GNPS also suggested 29 metabolites annotated through a molecular subnetwork. Some examples of these metabolites are shown in Figure 34 [73].

## 3. Bioactivity of Compounds Isolated from Sponges of the Genus *Phorbas*

The oceans are places on the Earth containing a wide spectrum of natural resources. The advent of new technologies allowed for profound study of marine biochemical diversity and the discovery of new bioactive marine natural products (MNPs). The complex habitats and exposure to extreme conditions of light, temperature, pH, salinity, and other external factors induce marine organisms to produce a wide variety of specific and potent active substances that cannot be found elsewhere [79]. The genus *Phorbas*, as well as several other sponges found in the aquatic environment, is a rich source of bioactive natural products such as alkaloids, terpenes, macrolides, steroids, and peptides.

Among the bioactivities described for compounds identified from the genus *Phorbas*, the cytotoxic activity (Table 1) stands out. However, other bioactivities (Table 2) have been reported. Bioactivity evaluation of pure compounds is often hampered by the low quantity that can be obtained from the natural source. Indeed, some compounds have been evaluated for their pharmacological properties only after being obtained on a larger scale by chemical synthesis.

### 3.1. Cytotoxic and Cytostatic Activity

Studies on the bioactivities of the metabolites isolated from sample of genus *Phorbas* mainly focus on the antiproliferative activity. For clarity and better reading purposes, data have been summarized in Table 1. 

The alkaloid zarzissine (**13**) showed a potent cytotoxic activity against three cell lines: murine leukemia P-388, human nasopharyngeal carcinoma KB, and human lung carcinoma NSCLC-N6 [37].

Macrolides such as phorbasides A (**20**), C (**22**), D (**23),** and E (**24**) exert prominent cytotoxic effects against HCT-116 (human colon cancer cell line), demonstrated through in vitro assays. However, phorbaside B (**21**) showed no activity. These results suggest that the presence of the free hydroxyl group at C-2 of the sugar moiety may play a key role in maintaining bioactivity [44]. Muironolide A (**19**) and phorboxazole A (**17**) are two other representative macrolides that possess cytotoxic activity against colon tumor cells [40]. 

Among steroids, phorbasterones A–D (**29**–**32**) displayed moderate cytotoxicity toward HCT-116 cells [48]. More recently, the lipid fraction obtained from samples of *P. amaranthus*, likely enriched of sterols, was found to possess antiproliferative properties against HCT-116 cells [73].

The sesterterpenoids phorbaketals A–C (**45**–**47**) exhibited cytotoxic activity against human colorectal cancer HT-29, hepatoma cancer HepG2, and adenocarcinoma human alveolar basal epithelial cells lines A549, while phorbaketal N (**50**) was cytotoxic against human renal cancer cell lines A498 and ACHN and pancreatic cancer cell line PANC-1. Phorbaketal N (**50**) showed a better activity than the positive control molecule, fluorouracil. Studies on **50** and derivatives may be a path in the search for new treatments for pancreatic cancer [52,53]. Phorbaketal H–I (**55**–**56**), isolated from the sponge *Monanchora* sp., showed weak cytotoxicity against the human renal A498 cancer cell line. Considering structure−activity relationships, a ketone group at C-5 of ring A of phorbaketals is much more favorable than a hydroxy group for activity, and the hydroperoxy group in the side chain is harmful to the cytotoxicity [54]. 

The compound phorbin A (**59**), isolated from *Monanchora* sp., a possible precursor of several sesterterpenoids isolated for *Phorbas*, also showed moderate activity against renal human cancer cell lines ACHN and A498, and potent cytotoxicity against human pancreatic cancer cell lines PANC-1 and MIA-paca, similar to or better than the positive control, 5-fluorouracil [54].

In addition, the sesterterpenoids isosuberitenone B (**84**) and 19-suberitenone B (**85**) unveiled significant grow-inhibitory effects against A549, HepG2, HT-29, and MCF-7 tumor cell lines. In the same study, compounds suberitenone B (**82**), oxaspirosuberitenone (**83**), and isooxaspirosuberitenone (**86**) showed moderate activity against these same cell lines. These sesterterpenoids isolated from *P. areolatus* were also tested against Mia-Paca-2 (pancreatic cancer cell line), but showed no activity [67]. 

Putative anticancer lead compounds with a diterpenoid backbone were a) phorbasin B–C (**88**–**89**) and the terpenyl-taurine phorbasin E (**91**), tested in a colon cancer model (HCT-116 cell line) [70] and b) gagunins A–Q (**101**–**117**) in K-562 cells (leukemia cell line) [75]. Among the latter, gagunins A and B (**101**–**102**) turned out to be the less active compounds. The authors suggest that the presence of a bulky group at C-11 of the five-membered ring negatively affects bioactivity, as compounds **107** and **108** are far less active than their congeners featuring either an acetoxyl group or hydrogen at the same position [75]. However, a synthetic gagunin A-derivative, in which the substituent groups placed on the three rings were replaced by hydroxyl groups, lacks activity [75]. 

The tetraterpenoid gukulenin B (**119**) exhibited significant cytotoxicity against human pharynx cell carcinoma line FaDu, gastric carcinoma cell MKN45, colon carcinoma cell line HCT-116, and renal carcinoma cell SN12C, and gukulenins C−F (**120**–**123**) showed potent cytotoxicity against K-562 and A549 cells [30]. Interestingly, gukulenin F (**123**) exhibited cytotoxicity against K-562 that was 17-fold more potent than doxorubicin, a positive control [30]. Moreover, gukulenin A (**118**) was shown to be a promising antitumor agent that (a) inhibited tumor growth in an ovarian cancer xenograft mouse model without any considerable adverse effect on their body weights, and (b) markedly reduced cell viability through apoptosis induction via the activation of caspases in four ovarian cancer cell lines. The cytotoxic activity of gukulenin A (**118**) is more potent than the positive control, cisplatin, in all ovarian cancer cells tested. This is the first report of an in vivo activity among compounds isolated from *Phorbas* [80]. 

Although several compounds showed promising results, cytotoxic studies on compounds from the genus *Phorbas* are, in most cases, at the initial phase. Only as recently as 2019 was there a study with gukulenin A (**118**) that advanced to in vivo studies using mouse models [80]. 

### 3.2. Other Biological Activities

Secondary metabolites isolated from sponges of the genus *Phorbas* displayed a large array of biological activities other than cytotoxicity (Table 2). 

Anchinopeptolides B–D (**2**–**4**), peptide alkaloids from *P. tenacior*, exhibited high efficacy in displacing specific ligands from their relevant receptors: human B2 bradykinin, which has a high correlation with inflammation mediators by causing vasodilation, increasing vascular permeability, and stimulating the synthesis of prostaglandins; neuropeptide Y, which is involved in physiological and homeostatic processes such as vasoconstriction and growth of fat tissue; and somatostatin receptors, which belong to the G protein class and have a wide expression pattern in both normal tissues and solid tumors [22,72,73]. On the other hand, anchinopeptolide A (**1**) was found to have weaker bioactivity in these binding assays [21]. The alkaloids zarzissine (**13**) and *p*-hydroxybenzaldehyde (**14**) showed slight antimicrobial activity against *Staphylococcus aureus* (gram-positive bacterium) and *C. albicans* and *C. tropicalis* (yeasts) [37]. 

The crude extract of *Phorbas topsenti* was reported to have high antioxidant activity in oxygen radical absorbance capacity (ORAC) assay, thereby leading to the isolation of phorbatopsins A–C (**14**–**16**), i.e., the compounds responsible for the observed radical scavenging activity. The antioxidant capacity of the isolated compounds was also evaluated with ORAC assay, measuring the loss of fluorescence of fluorescein in the presence of the oxidative species AAPH [2,2′-azobis(2-amidino-propane) dihydrochloride] and compared with Trolox^®^, used as the positive control. Phorbatopsin A (**14**) was the most active substance, with an ORAC value comparable to Trolox®. These data clearly indicate the importance of the C5–C6 double bond in compound **14** in improving the antioxidant properties of the phorbatopsin scaffold [26]. 

Macrolides phorboxazoles A–B (**17**–**18**) exhibited antifungal activity in the agar disc diffusion inhibition assay against *Candida albicans* and *Saccharomyces carlsbergensis* [24]. Another example is the macrolide muironolide A (**19**), which was reported to have antifungal activity against strains of *Cryptococcus neoformans* [81].

The genus *Phorbas* is also a source of other bioactive compounds, such the steroids amaroxocanes A–B (**37**–**38**), which were isolated and tested for chemical defense of the Caribbean coral reef sponge *Phorbas amaranthus* from fish predators. Amaroxocane B (**38**) showed significant deterrent activity (3/10 pellets eaten), while amaroxocane A (**37**) elicited little feeding deterrence (8/10 pellets eaten) against a common reef predator, namely the bluehead wrasse. This study suggests that structural differences in the heterocycle moiety or the degree of sulfation may be responsible for differential anti-predatory activity [50].

Phorbaketal A (**45**), which also showed cytotoxic activity, can promote osteogenic differentiation of human mesenchymal stem cells, which exhibited increased levels of differentiation markers such as osteocalcin, Dlx5, ALP, Runx2, and TAZ after drug exposure. This compound showed potential for bone reformation processes and new anabolic therapeutics in bone diseases. Moreover, as inhibiting mesenchymal stem cells differentiate into adipocytes through a transcriptional coactivator with PDZ-binding motif, compound **45** may be a promising lead in designing novel drugs to treat obesity. In addition, this compound showed a promising dose dependent inhibition of inflammatory mediators via down-regulation of the NF-κB pathway and up-regulation of the HO-1 pathway [82,83,84]. The sesterterpenoids phorbasones A–B (**76**–**77**) promote calcium deposition in mensenchymal C3H10T1/2 cells, thus inducing osteoblast differentiation. The authors concluded that phorbasone A (**76**) showed a distinct calcium deposition effect as compared to phorbasone B (**77**). Particularly, gene expression analysis of osteoblast differentiation markers revealed that compound **76** increases Runx2 (a Runt protein), ALP (alkaline phosphatase), OSX (osterix), PTH (parathyroid hormone), and PTHrP (PTHrelated peptide) mRNA [65]. Another study reported on the potent inhibitory activity on nitric oxide (NO) production in RAW 264.7 LPS-activated mouse macrophage cells by phorbasone A acetate (**79**). This result indicated that effective suppression of NO production is a valuable strategy for the discovery of anti-inflammatory compounds [65]. 

Among sesterterpenoids, suberitenones A and B, oxaspirosuberitenone, isosuberitenone B, 19-episuberitenone B, and isooxaspirosuberitenone (**81**–**86**), isolated from *Phorbas areolatus* (non-polar fraction), were tested against gram positive (methicillin resistant and methicillin sensitive *Staphylococcus aureus*, MRSA, and MSSA) and gram negative (*Escherichia coli*, and *Klebsiella pneumoniae*) bacteria. This study reported oxaspirosuberitenone (**83**) as a significant antimicrobial compound against MRSA at the highest concentration tested [67,85].

Ansellone A (**65**) can activate cAMP signaling in HEK293 cells, derived from human embryonic kidney cells grown in a tissue culture, which is a very important technique for the development of treatments for several diseases such as heart failure, cancer, and neurodegenerative diseases. cAMP signaling activation by ansellone A (**65**) was comparable to that of forskolin, a natural product used for the treatment of cancer, obesity, and allergies [56]. The latency reversal activity (LRA) of **65**, which has the function of reactivating the virus production in infected cells and producing an immune response or cell death, was also reported and determined by quantification of the changes in intracellular GFP expression in microplate [64]. The sesterterpenoid ansellone B (**66**) was reported as a potent inhibitor on nitric oxide production in RAW 264.7 LPS-activated mouse macrophage cells [59]. 

Alotaketals A and B (**60**–**61**) have also been reported for the activation of the cAMP cell signaling pathway. In addition, the compounds alotaketal C (**62**) and D (**63**) and anvilone A (**74**) were reported to activate the latent proviral HIV-1 gene expression. Notably, alotaketal C (**62**) was more potent and gave a stronger effect than the control compound prostratin at the same concentration, while alotaketal D (**63**) and anvilone A (**74**) elicited similar responses as prostratin [52,54,77,78]. The diterpen phorbasin H (**94**) was reported as an inhibitor of the yeast-to-hypha transition in *Candida albicans.* Growth experiments suggested that this compound does not inhibit yeast cell growth but inhibits filamentous growth in *C. albicans,* which means that the phorbasin H (**94**) induces a change in *C. albicans* morphology [86]. Another study reported the ethanolic extract rich in phorbasins (**87**–**89**) from the *Phorbas* sp. could exert growth inhibitory activity against gram positive bacteria, such as *Staphylococcus aureus* and *Micrococcus luteus*. It was not possible to test pure compounds due to the low amount available and their instability [31,68]. 

One study concerning the cosmetic use of gagunin D (**104**) identified this compound as a potential anti-melanogenic agent. Gagunin D (**104**) inhibited the synthesis of melanin in both mouse melan-a cells and a reconstructed human skin model. Suppression of tyrosinase expression and increased rate of tyrosinase degradation as well as inhibition of its enzymatic activity are putative mechanisms underlying the anti-melanogenic activity exhibited by gagunin D (**104**). These studies highlight the potential use of gagunin D (**104**) for skin lightening cosmetic formulations [87].

The summary of these biological activities reported for compounds isolated from extracts of the genus *Phorbas* sp. are found in Table 2.

As can be seen, most of the assays were carried out in vitro and obtained very promising results. Thus, more robust studies and in vivo assays of these substances must be carried out to prove their effectiveness against the various diseases previously tested in these studies. 

## 4. Conclusions

Marine sponges, including *Phorbas*, still represent a prolific source of new molecules yet to be discovered. Novel and more powerful tools should be developed to (a) ameliorate and accelerate the discovery process and (b) reduce the risk of re-discovery of MNPs. Dereplication based upon liquid chromatography coupled with high-resolution tandem mass spectrometry is a well-suited approach to solve these issues and allow detection of new metabolites, even from well-known sponges such as *Phorbas*. In this regard, molecular networking [89,90] could represent a suitable means for (a) fast detection, annotation, and visualization of known compounds and their novel analogues and (b) an in-depth re-examination of *Phorbas* species to unlock overlooked chemical entities. A fundamental aspect in bioactive natural products research is certainly the assignment of stereochemistry to identify the pharmacophore of a molecule and investigate drug–target interaction. Due to the limited amounts of available compound, elucidation of the stereochemistry of natural products is very challenging. Therefore, as shown for many metabolites from natural sources, including *Phorbas* sponges, chemical degradation of a molecule into simpler compounds can be exploited as a valuable approach to assist spectroscopic analysis in the assignment of relative and absolute configuration of natural products.

This article provides a comprehensive review of the literature on sponges of the genus *Phorbas,* throughout 1993–2020, and summarizes the discovery of one hundred and thirty-two compounds, including alkaloids, macrolides, terpenoids, and steroids, and a brief insight into the putative biogenetic pathway and biosynthetic origin of sesterterpenoids from *Phorbas* sponges. Moreover, this review includes a survey on pharmacological activities shown by the reported metabolites. 

Cytotoxic activity displayed by secondary metabolites from this genus make them interesting MNPs for development of new drugs with antineoplastic activity. Thus, this article aims to be useful for the bioprospecting process of marine sponges of the genus *Phorbas* and to bring attention to its biochemical diversity.

## Figures and Tables

**Table 1:** List of MNPs isolated from *Phorbas* sp. having antiproliferative activity.

	Name	Class	Species	Cell Lines	Dose/Concentration	Reference
**13**	Zarzissine	Alkaloid	*Phorbas* *tenacior*	P-388 ^a^	IC_50_ 12 µg/mL	[37]
				KB ^b^	IC_50_ 5 µg/mL	
				NSCLC-N6 ^c^	IC_50_ 10 µg/mL	
**17**	Phorboxazole A	Macrolide	*Phorbas* sp.	HCT-116 ^d^	GI_50_ 4.36 × 10^−10^ M	[24]
				HT29 ^d^	GI_50_ 3.31 × 10^−10^ M	
**19**	Muironolide A	Macrolide	*Phorbas* sp.	HCT-116 ^d^	IC_50_ 96.5 μg/mL	[40]
**20**	Phorbaside A	Macrolide	*Phorbas* sp.	HCT-116 ^d^	IC_50_ 30.0 μM	[44]
**22**	Phorbaside C	Macrolide	*Phorbas* sp.	HCT-116 ^d^	IC_50_ 2 μM	[44]
**23**	Phorbaside D	Macrolide	*Phorbas* sp.	HCT-116 ^d^	IC_50_ 61.9 μM	[44]
**24**	Phorbaside E	Macrolide	*Phorbas* sp.	HCT-116 ^d^	IC_50_ 10.2 μM	[44]
**29**	Phorbasterone A	Steroid	*Phorbas* *amaranthus*	HCT-116 ^d^	IC_50_ 1–3 µg/mL	[48,73]
**30**	Phorbasterone B	Steroid	*Phorbas* *amaranthus*	HCT-116 ^d^	IC_50_ 1–3 µg/mL	[48,73]
**31–32**	Phorbasterone C	Steroid	*Phorbas* *amaranthus*	HCT-116 ^d^	IC_50_ 1–3 µg/mL	[48,73]
**33–34**	Phorbasterone D	Steroid	*Phorbas* *amaranthus*	HCT-116 ^d^	IC_50_ 1–3 µg/mL	[48,73]
**45**	Phorbaketal A	Sesterterpenoid	*Phorbas* sp.	A549 ^c^	IC_50_ 11–12 µg mL^−1^	[52,54]
				HT-29 ^d^	IC_50_ 11–12 µg mL^−1^	
				HepG2 ^e^	IC_50_ 11–12 µg mL^−1^	
**46**	Phorbaketal B	Sesterterpenoid	*Phorbas* sp.	A549 ^c^	IC_50_ 12–460 µg/mL	[52,54]
				HT-29 ^d^	IC_50_ 12–460 µg/mL	
				HepG2 ^e^	IC_50_ 12–460 µg/mL	
**47**	Phorbaketal C	Sesterterpenoid	*Phorbas* sp.	A549 ^c^	IC_50_ 12–460 µg/mL	[52,54]
				HT-29 ^d^	IC_50_ 12–460 µg/mL	
				HepG2 ^e^	IC_50_ 12–460 µg/mL	
				HT-29 ^d^	LG_50_ 5–15 μM	
**50**	Phorbaketal N	Sesterterpenoid	*Phorbas* sp.	PANC-1 ^f^	IC_50_ 11.4 µM	[53]
				A498 ^g^	IC_50_ 18.7 µM	
				ACHN ^g^	LC_50_ 24.4 µM	
**84**	Isosuberitenone B	Sesterterpenoid	*Phorbas* *areolatus*	A549 ^c^	IC_50_ 8.8 μM	[67]
				HT-29 ^d^	IC_50_ 9.0 μM	
				HepG2 ^e^	IC_50_ 7.4 μM	
				MCF-7 ^h^	IC_50_ 8.8 μM	
**85**	19-episuberitenone B	Sesterterpenoid	*Phorbas* *areolatus*	A549 ^c^	IC_50_ 5.1 μM	[67]
				HT-29 ^d^	IC_50_ 6.4 μM	
				HepG2 ^e^	IC_50_ 5.0 μM	
				MCF-7 ^h^	IC_50_ 5.1 μM	
**88**	Phorbasin B	Diterpene	*Phorbas* sp.	A549 ^c^	LG_50_ 5–15 μM	[70]
				HT-29 ^d^	LG_50_ 5–15 μM	
**89**	Phorbasin C	Diterpene	*Phorbas* sp.	A549 ^c^	LG_50_ 5–15 μM	[70]
				HT-29 ^d^	LG_50_ 5–15 μM	
**91**	Phorbasin E	Terpenyl-taurine	*Phorbas* sp.	A549 ^c^	LG_50_ 5–15 μM	[70]
				HT-29 ^d^	LG_50_ 5–15 μM	
**101**	Gagunin A	Diterpenoid	*Phorbas* sp.	K-562 ^a^	LC_50_ 50.1 µg/mL	[75]
**102**	Gagunin B	Diterpenoid	*Phorbas* sp.	K-562 ^a^	LC_50_ 10.4 µg/mL	[75]
**103**	Gagunin C	Diterpenoid	*Phorbas* sp.	K-562 ^a^	LC_50_ 0.71 µg/mL	[75]
**104**	Gagunin D	Diterpenoid	*Phorbas* sp.	K-562 ^a^	LC_50_ 0.13 µg/mL	[75]
**105**	Gagunin E	Diterpenoid	*Phorbas* sp.	K-562 ^a^	LC_50_ 0.03 µg/mL	[75]
**106**	Gagunin F	Diterpenoid	*Phorbas* sp.	K-562 ^a^	LC_50_ 0.11 µg/mL	[75]
**107**	Gagunin G	Diterpenoid	*Phorbas* sp.	K-562 ^a^	LC_50_ 2.0 µg/mL	[75]
**108**	Gagunin H	Diterpenoid	*Phorbas* sp.	K-562 ^a^	LC_50_ 10.0 µg/mL	[76]
**109**	Gagunin I	Diterpenoid	*Phorbas* sp.	K-562 ^a^	LC_50_ 11.5 µg/mL	[76]
**110**	Gagunin J	Diterpenoid	*Phorbas* sp.	K-562 ^a^	LC_50_ 9.1 µg/mL	[76]
**111**	Gagunin K	Diterpenoid	*Phorbas* sp.	K-562 ^a^	LC_50_ 17.5 µg/mL	[76]
**112**	Gagunin L	Diterpenoid	*Phorbas* sp.	K-562 ^a^	LC_50_ 12.5 µg/mL	[76]
**113**	Gagunin M	Diterpenoid	*Phorbas* sp.	K-562 ^a^	LC_50_ 0.71 µg/mL	[76]
**114**	Gagunin N	Diterpenoid	*Phorbas* sp.	K-562 ^a^	LC_50_ > 50 µg/mL	[76]
**115**	Gagunin O	Diterpenoid	*Phorbas* sp.	K-562 ^a^	LC_50_ 11.1 µg/mL	[76]
**116**	Gagunin P	Diterpenoid	*Phorbas* sp.	K-562 ^a^	LC_50_ 8.5 µg/mL	[76]
**117**	Gagunin Q	Diterpenoid	*Phorbas* sp.	K-562 ^a^	LC_50_ > 50 µg/mL	[76]
**118**	Gukulenin A	tetraterpenoid	*Phorbas* *gukulensis*	HCT-116 ^d^	IC_50_ 62 nM	[30]
				FaDu ^b^	IC_50_ 57 nM	
				SN12C ^g^	IC_50_ 92 nM	
				MKN45 ^j^	IC_50_ 0.13 nM	
				TOVG-21G ^i^	IC_50_ 0.04 μM	[80]
				OVCAR-3 ^i^	IC_50_ 0.13 μM	
				A2780 ^i^	IC_50_ 0.03 μM	
				SKOV3 ^i^	IC_50_ 0.36 μM	
**119**	Gukulenin B	tetraterpenoid	*Phorbas* *gukulensis*	HCT-116 ^d^	IC_50_ 0.55 μM	[30]
				A2780 ^i^		
				FaDu ^b^	IC_50_ 0.63 μM	
				SN12C ^g^	IC_50_ 0.61 μM	
				MKN45 ^j^	IC_50_ 0.72 μM	
**123**	Gukulenin F	Tetraterpenoid	*Phorbas* *gukulensis*	K-562 ^a^	LC_50_ 0.4 µM	[30]
				FaDu ^b^	IC_50_ 0.63 μM	
				SN12C ^g^	IC_50_ 0.61 μM	
				MKN45 ^j^	IC_50_ 0.72 μM	

^a^ leukemia; ^b^ pharynx carcinoma; ^c^ lung carcinoma; ^d^ colon carcinoma; ^e^ liver carcinoma; ^f^ pancreas carcinoma; ^g^ kidney carcinoma; ^h^ breast carcinoma; ^i^ ovarian cancer; ^j^ gastric cancer.

**Table 2:** List of MNPs originated from *Phorbas* with biological activities.

	Compound	Class	Species	Biological Activity	Dose/Concentration	Reference
**1**	Anchinopeptolide A	Alkaloid	*P. tenacior*	Displacement of specific ligands from their biochemical receptors	5 µg/mL-average inhibition values roughly 35–40% in all receptor binding	[21,22]
**2**	Anchinopeptolide B	Alkaloid	*P. tenacior*	Displacement of specific ligands from their biochemical receptors	5 µg/mL-71% human B2 bradykinin receptor; 80% neuropeptide Y receptor	[21,22]
**3**	Anchinopeptolide C	Alkaloid	*P. tenacior*	Displacement of specific ligands from their biochemical receptors	5 µg/mL-62% somatostatin receptor; 52% human B2 bradykinin receptor; 57% neuropeptide Y receptor	[21,22]
**4**	Anchinopeptolide D	Alkaloid	*P. tenacior*	Displacement of specific ligands from their biochemical receptors	5 µg/mL-77% somatostatin receptor	[21,22]
**13**	Zarzissine	Alkaloid	*P. topsenti*	Antimicrobial	Paper disk agar-(100 µg, purified product) 12,10, and 11 mm	[37]
**14**	*p*-Hydroxybenzaldehyde	Alkaloid	*P. topsenti*	Antimicrobial	Paper disk agar-(100 µg, purified product) 8,7, and 7 mm	[37]
**14**	Phorbatopsin A	Alkaloid	*P. topsenti*	Antioxidant	ORAC_FL_ 0.88 ± 0.28	[26]
**15**	Phorbatopsin B	Alkaloid	*P. topsenti*	Antioxidant	ORAC_FL_ 0.50 ± 0.08	[26]
**16**	Phorbatopsin C	Alkaloid	*P. topsenti*	Antioxidant	ORAC_FL_ 0.21 ± 0.02	[26]
**17**	Phorboxazole A	Macrolide	*Phorbas* sp.	Antifungal	Agar disk diffusion assay-*C. albicans*: 12 mm (1 µg) and 9 mm (0.1 µg); *Saccharomyces carlsbergensis*: 1, 20 mm (1 µg), and 13 mm (0.1 µg)	[24]
**18**	Phorboxazole B	Macrolide	*Phorbas* sp.	Antifungal	Agar disk diffusion assay-C. albicans: 11 mm (1 µg) and 8 mm (0.1 µ g); *Saccharomyces carlsbergensis*: 1, 16 mm (1 µg), and 10 mm (0.1 µg)	[24]
**19**	Muironolide A	Macrolide	*Phorbas* sp.	Antifungal	MIC 16 μg/mL	[40]
**22**	Phorbaside C	Macrolide	*Phorbas* sp.	Macrophage infectivity potentiator (Mip)	Binding affinity of 75 with Chlamydia pneumoniae	[88]
**37**	Amaroxocane A	Steroid	*P.amaranthus*	Anti-predatory activity	Little feeding deterrence (8/10 pellets eaten)	[50]
**38**	Amaroxocane B	Steroid	*P. amaranthus.*	Anti-predatory activity	Significant deterrent activity (3/10 pellets eaten)	[50]
**45**	Phorbaketal A	Sesterterpenoid	*Phorbas* sp.	Osteogenic differentiation Anti-inflammatory	Phorbaketal A stimulates TAZ-mediated osteoblast differentiation through the activation of extracellular signal-regulated kinase (1–10 µg/mL) Dose dependent inhibition of LPS-induced production of inflammatory cytokines and the transcriptional activity NF-κB (2.5, 5, and 10 μM) and adipocyte differentiation through transcriptional coactivator with PDZ-binding motif (1–10 µg/mL)	[84] [82,83]
**60**	Alotaketal A	Sesterterpenoid	*Phorbas* sp.	cAMP signaling activation	cAMP cell signaling pathway-EC_50_ of 18 nM	[55]
**61**	Alotaketal B	Sesterterpenoid	*Phorbas* sp.	cAMP signaling activation	cAMP cell signaling pathway-EC_50_ of 240 nM	[55]
**62**	Alotaketal C	Sesterterpenoid	*Phorbas* sp.	Latency-reversing agent (LRA)	HIV-1 provirus/GFP expression of J-Lat 9.2 cells-1 μM	[56,57,78]
**63**	Alotaketal D	Sesterterpenoid	*Phorbas* sp.	Latency-reversing agent (LRA)	HIV-1 provirus/GFP expression of J-Lat 9.2 cells-30 μM	[56,57,78]
**65**	Ansellone A	Sesterterpenoid	*Phorbas* sp.	cAMP signaling activation Latency-reversing agent (LRA)cAMP activator	cAMP cell signaling pathway-EC_50_ of 14 µM HIV-1 provirus/GFP expression of J-Lat 9.2 cells-30 μM	[57,62]
**66**	Ansellone B	Sesterterpenoid	*Phorbas* sp.	Inhibition of inducible NOS (iNOS)	RAW 264.7 LPS-activated mouse macrophage cells-IC_50_ = f 4.5 μM,	[66]
**73**	Anvilone A	Sesterterpenoid	*Phorbas* sp.	Latency-reversing agent (LRAs)	HIV-1 provirus/GFP expression of J-Lat 9.2 cells-30 μM	[57]
**76**	Phorbasone A	Sesterterpenoid	*Phorbas* sp.	Osteogenic properties	Calcium deposition effect at a concentration of 0.5 μg/mL	[65]
**79**	Phorbasone A acetate	Sesterterpenoid	*Phorbas* sp.	Inhibition of inducible NOS (iNOS)	Inhibitory activity on NOS in RAW 264.7 LPS-activated mouse macrophage cells-IC_50_ = 2.8 μM	[66]
**83**	Oxaspirosuberitenone	Sesterterpenoids	*P. areolatus*	Antimicrobial	Activity against MRSA at the highest concentration tested (160 µM)	[67]
**94**	Phorbasin H	Diterpenoid	*Phorbas* sp.	Antifungal	Suppression of the hyphal development of *C. albicans* (250 μg/mL)	[86]
**93–95**	Phorbasins	Diterpenoid	*Phorbas* sp.	Antifungal	EtOH extract-growth inhibitory activity against the gram-positive bacteria *Staphylococcus aureus* and *Micrococcus luteus*–Concentration: N/A	[68,69]
**104**	Gagunin D	Diterpenoid	*Phorbas* sp.	Anti-melanogenic	IC_50_ = 5.7 µg/mL; 10 µM on UVB irradiated human skin models demonstrated a considerable reduction melanin biosynthesis	[87]
**101–117**	Gagunins	Diterpenoid	*Phorbas* sp.	Isocitrate lyase (ICL) inhibition	LC_50_ of 55–140 µg/mL	[76]
**125**	Astaxanthin	Carotenoid	*P. topsenti*	Antioxidant	ORAC_FL_ 0.22 ± 0.02	[26]
**126**	Adonirubin	Carotenoid	*P. topsenti*	Antioxidant	ORAC_FL_ 0.024 ± 0.001	[26]
**127**	Taurine	Sulfonic acid	*P. topsenti*	Antioxidant	ORAC_FL_ 0.083 ± 0.013	[26]
**128**	Taurobetaine	Sulfonic acid	*P. topsenti*	Antioxidant	ORAC_FL_ 00.019 ± 0.002	[26]
